# IFN-β Deficiency Results in Fatal or Demyelinating Disease in C57BL/6 Mice Infected With Theiler’s Murine Encephalomyelitis Viruses

**DOI:** 10.3389/fimmu.2022.786940

**Published:** 2022-02-09

**Authors:** Melanie Bühler, Sandra Runft, Dandan Li, Jasper Götting, Claudia N. Detje, Vanessa Nippold, Melanie Stoff, Andreas Beineke, Thomas Schulz, Ulrich Kalinke, Wolfgang Baumgärtner, Ingo Gerhauser

**Affiliations:** ^1^ University of Veterinary Medicine Hannover, Foundation, Hannover, Germany; ^2^ Institute of Virology, Hannover Medical School, Hannover, Germany; ^3^ Institute for Experimental Infection Research, Twincore, Centre for Experimental and Clinical Infection Research, a Joint Venture between the Hannover Medical School and the Helmholtz Centre for Infection Research, Hannover, Germany

**Keywords:** demyelination, encephalitis, immunohistochemistry, IFN-β, knockout mice, RT-qPCR, TMEV strains

## Abstract

*Type I Interferons* (IFN-I) are important inducers of the antiviral immune response and immune modulators. IFN-β is the most highly expressed IFN-I in the *central nervous system* (CNS). The infection of SJL mice with the BeAn or the DA strain of *Theiler’s murine encephalomyelitis virus* (TMEV) results in a progressive demyelinating disease. C57BL/6 mice are usually resistant to TMEV-induced demyelination and eliminate these strains from the CNS within several weeks. Using C57BL/6 *IFN-β knockout* (IFN-β^-/-^) mice infected with TMEV, we evaluated the role of IFN-β in neuroinfection. Despite the resistance of C57BL/6 *wild type* (WT) mice to TMEV infection, DA-infected IFN-β^-/-^ mice had to be killed at 7 to 8 *days post infection* (dpi) due to severe clinical disease. In contrast, BeAn-infected IFN-β^-/-^ mice survived until 98 dpi. Nevertheless at 14 dpi, BeAn-infected IFN-β^-/-^ mice showed a stronger encephalitis and astrogliosis, higher viral load as well as higher mRNA levels of *Isg15*, *Eif2ak2* (PKR), *Tnfa*, *Il1b*, *Il10*, *Il12* and *Ifng* in the cerebrum than BeAn-infected WT mice. Moreover, the majority of IFN-β^-/-^ mice did not clear the virus from the CNS and developed mild demyelination in the spinal cord at 98 dpi, whereas virus and lesions were absent in the spinal cord of WT mice. Persistently infected IFN-β^-/-^ mice also had higher *Isg15*, *Eif2ak1*, *Tnfa*, *Il1a*, *Il1b* and *Ifng* mRNA levels in the spinal cord at 98 dpi than their virus-negative counterparts indicating an activation of IFN-I signaling and ongoing inflammation. Most importantly, BeAn-infected NesCre^+/-^ IFN-β^fl/fl^ mice, which do not express IFN-β in neurons, astrocytes and oligodendrocytes, only developed mild brain lesions similar to WT mice. Consequently, IFN-β produced by neuroectodermal cells does not seem to play a critical role in the resistance of C57BL/6 mice against fatal and demyelinating disease induced by TMEV strains.

## Introduction

*Theiler’s murine encephalomyelitis virus* (TMEV) belongs to the family of *Picornaviridae* and is used to study long-term consequences caused by viral encephalitis (1). The intracerebral inoculation of *Daniel’s* (DA) and *BeAn 8386* (BeAn) strains of TMEV into susceptible SJL mice results in acute polioencephalomyelitis and chronic progressive demyelinating leukomyelitis, whereas resistant *C57BL/6* (B6) mice usually clear the virus from the *central nervous system* (CNS) (1). The DA strain causes more severe brain inflammation in the acute phase of the disease than the BeAn strain and even induces seizures and epilepsy in B6 mice (2, 3). Resistance of B6 mice to *TMEV-induced demyelinating disease* (TMEV-IDD) is associated with the induction of robust innate immune responses (1, 4), which is partly linked to the H-2D region of the *major histocompatibility complex* (MHC) class I gene (5), and lower levels of anti-inflammatory cytokines such as *transforming growth factor* (TGF)-β and *interleukin* (IL)-10 (3, 6, 7).

The TMEV *leader* (L) protein is a 76 amino acid protein cleaved off the N terminus of the viral polyprotein and contributes to viral persistence (8, 9). The L protein can interfere with cellular mRNA export from the nucleus and *interferon regulatory factor* (IRF)-3 dimerization, which inhibits *type I interferon* (IFN-I) gene transcription enhancing viral replication and reduces TMEV-induced hippocampal injury (8, 10–12). Moreover, the L* protein, encoded by an alternative open reading frame, blocks the antiviral OAS/RNase L pathway through direct interaction with the ankyrin domain of RNase L (13). The *IFN-I receptor* (IFNAR) is located on the cell surface and composed of the subunits IFNAR1 and IFNAR2. IFN-I signal transduction is mediated by *Janus kinase* (JAK)-1 and *tyrosine kinase* (TYK)-2, which activate *interferon stimulated gene factor* (ISGF)-3. This transcription factor is a ternary complex consisting of *signal transducers and activator of transcription* (STAT)-1 and -2 and IRF-9, which binds to the *IFN stimulated response element* (ISREs) in the promoter region of over 300 *interferon stimulated genes* (ISGs) (14). ISG15, *protein kinase R* (PKR), 2’-5’-*oligoadenylatsynthetase* (OAS) and *myxovirus resistance* (Mx) proteins represent classical antiviral effectors, which are induced in multiple vertebrate species and block all steps of viral replication (14, 15). IFNAR knockout mice develop a rapid fatal encephalitis after TMEV infection demonstrating the essential role of ISGs in virus control (16). Moreover, C57BL/6 mice exhibit a higher expression of ISG15 and PKR in the spinal cord compared to SJL mice, which likely contributes to virus elimination and their resistance to TMEV-IDD (4).

Under noninflammatory conditions the CNS is characterized by low constitutive IFN-β expression, whereas upon infection with neurotropic viruses strong IFN-β responses are induced within the brain (17). IFN-β^-/-^ mice are highly susceptible to vaccinia virus (*Poxviridae*) (18), *La Crosse virus* (LACV, *Bunyaviridae*) (19), *coxsackievirus B3* (CVB3, *Picornaviridae*) (18), *vesicular stomatitis virus* (VSV, *Rhabdoviridae*) (20), *West Nile virus* (WNV, *Flaviviridae*) (21) and influenza A viruses (IAV, *Orthomyxoviridae*) (22), but not to a low neurovirulent strain of Sindbis virus (*Togaviridae*) (23). IFN-β triggers IFNAR signaling necessary for early restriction of viral replication and spread (17, 23). IFN-β also induces the synthesis of the transcription factor IRF-7 and thus facilitates the production of most IFN-α subtypes (24), whereas IFN-β deficiency does not seem to impair IFN-α production in the CNS (23). The role of IFN-β in the pathogenesis of neuroinflammatory processes caused by TMEV infection has not been determined so far. Consequently, the aim of the present study was to analyze the effect of IFN-β deficiency on the disease course after TMEV infection.

## Material and Methods

### Animals Experiment

5 to 6-week old IFN-β^-/-^ mice (B6.129P2-Ifnb^tm1 (Lambda2 (315)) GBF^) mice (25) and wild type C57BL/6 mice (Charles River Laboratories) were intracerebrally infected with 1x10^5^ plaque-forming units (PFU) TMEV-BeAn or TMEV-DA (2, 26, 27). Clinical examination included weekly clinical scoring and RotaRod^®^ performance tests (26, 28). Necropsy was performed to obtain frozen and paraffin-embedded brain and spinal cord samples after perfusion with phosphate buffered saline.

For *in vivo* imaging of IFN-β expression, 5 to 6-week old luciferase reporter mice (IFN-β^Δβluc/wt^) backcrossed to a C57BL/6 albino background were intracerebrally infected with 1x10^5^ PFU TMEV-BeAn or TMEV-DA or received an intracerebral injection of cell culture medium (Dulbecco’s Modified Eagle Medium, DMEM, PAA Laboratories) as control. Mice were injected with 150 mg/kg of D-luciferin in PBS (Perkin-Elmer) into the lateral tail vein, anesthetized with 2.5% Isofluran (Abbot), and monitored using an IVIS Spectrum CT (Perkin-Elmer) at 0, 6, 12, 24, 48 and 72 hours post infection/injection. Photon flux was quantified using the Living Image 4.3.1 software.

To generate tissue-specific IFN-β^-/-^ mice, IFN-β^floxβ-luc/floxβ-luc^ animals were intercrossed with NesCre^+/-^ mice that express Cre in neuroectodermal cells (neurons, astrocytes and oligodendrocytes). 5 to 6-week old NesCre^+/-^ IFN-β^fl/fl^ mice (B6.Cg-Tg (Nes-Cre) 1Kln-Ifnb^tm2.1(luc)lien^) and wild type littermates (NesCre^-/-^ IFN-β^fl/fl^ mice) were intracerebrally infected with 1x10^5^ PFU TMEV-BeAn.

### Virus Sequencing and Plaque Size Analysis

Nucleic acid was extracted and sequenced as described (29). Briefly, RNA from cell culture supernatant was recovered using a NucleoSpin kit (Macherey-Nagel) and RNA sequencing libraries were prepared using the ScriptSeq v2 RNA-Seq chemistry (Illumina). Libraries were sequenced on an Illumina MiSeq using a 500v2 kit generating approximately 2 million paired-end reads of 250 bp length per sample. TMEV genomes were constructed by combining the *de novo* assembly generated in CLC Genomics Workbench (Qiagen) and the JX443418 mapping consensus in Geneious Prime 2022 (Biomatters). Assembled genomes and GenBank sequences were aligned using MAFFT v7.450 and a Neighbor-Joining tree was constructed in Geneious Prime 2022 using default parameters and 1,000 bootstrap replicates. The following GenBank accession numbers were used for the phylogeny: NC_001366, NC_009448, JX443418, M16020, M20301, M20562, KF680264, MK343442, MK343443, X56019, HQ652539, EU718732, EU718733, EU723238.

Plaque assay was performed as described (30). Briefly, cerebral tissue was weighed, diluted in DMEM to a concentration of 10% and homogenized using Omni Tissue Homogenizer (Süd-Laborbedarf GmbH). Serial dilutions of homogenates were added to 6-well culture plates (Sigma-Aldrich) of confluent L cells for 1 hour at room temperature. Subsequently, cells were covered with methyl cellulose (Sigma-Aldrich) and after an incubation for 72 hours at 37°C cells were fixed with 10% buffered formalin. PFU/ml were determined after staining with crystal violet (Merck). Plaque sizes were evaluated using photos of culture plates and analysis software (analySIS 3.1 software package; Soft Imaging system).

### Histology and Immunohistochemistry

2-4 µm paraffin section of the brain and spinal cord were stained with hematoxylin and eosin and used for histological examination. Perivascular mononuclear infiltrates were semiquantitatively quantified (0: no infiltrates; 1: one layer of infiltrates; 2: 2-3 layers of infiltrates; 3: > 3 layers of infiltrates) in different areas of the cerebrum (meninges, cortex cerebri, subcortical white matter, hippocampus, thalamus/hypothalamus, third ventricle, lateral ventricles and basal ganglia) separately in both cerebral hemispheres (16 values per animal) and the mean calculated for each mouse. Neuronal cell loss was evaluated separately in the left and right hippocampus using a semiquantitative scoring system (0: no cell loss; 1: <25% of neurons lost; 2: 25-50% of neurons lost; 3: >50% of neurons lost) and the mean of both values determined. Moreover, the cellularity in the spinal cord was evaluated semiquantitatively (0: normal; 1: mildly increased; 2: moderately increased; 3: severely increased) in the dorsal left, dorsal right, ventral left and ventral right quarters of the gray and white matter of the cervical, thoracic and lumbar spinal cord (24 values per animal) and the mean calculated for each mouse.

Immunohistochemistry of paraffin-embedded brain sections was performed to detect TMEV antigen (1:2000) (31), CD3^+^ T cells (GA50361-2; Agilent Dako; 1:500), CD45R^+^ B cells (553085; BD Biosciences; 1:1000), Iba-1^+^ macrophages (019-19741; Wako Chemicals GmbH; 1:1000) and GFAP (GA52461-2; Agilent Dako; 1:1000) (32). Morphometry of immunohistochemically stained slides was used to quantify the infiltration of CD3^+^ T cells, CD45R^+^ B cells and Iba-1^+^ macrophages (percentage of immunopositive area) into the brain at two section levels (cerebrum with hippocampus, thalamus and hypothalamus; cerebellum with medulla oblongata) using a digital microscope (HS All-in-one Fluorescence Microscope BZ-9000 Generation II, BIOREVO, KEYENCE Deutschland GmbH) and analysis software (analySIS 3.1 software package; Soft Imaging system). Viral load was evaluated using a semiquantitative scoring system (0: no TMEV^+^ cells; 1: <25% TMEV^+^ cells; 2: 25-50% TMEV^+^ cells; 3: >50% TMEV^+^ cells) in different areas of the cerebrum (see above) and the mean calculated. Astrogliosis was also evaluated semiquantitatively (0: no; 1: mild; 2: moderate; 3: severe) in these areas of the cerebrum (32). In the spinal cord the absolute number of TMEV-infected cells was determined in three complete transversal sections (cervical, thoracic, lumbar).

### Fluorescent *In Situ* Hybridization

To localize IFN-β expression in the brain, *fluorescent in situ hybridization* (FISH) was performed using an RNA probe (Ifnb1, 63037-06; Affymetrix) and the ViewRNA ISH Tissue Assay Kit (Thermo Fisher Scientific) as well as the ViewRNA Chromogenic Signal Amplification Kit (Thermo Fisher Scientific) as described (33, 34). Briefly, paraffin sections were deparaffinized, boiled for 10 min in pretreatment solution, incubated with protease QF^®^ for 20 min and hybridized with the RNA probe for 6 h at 40°C. Following amplification steps, phase contrast and Cy3 fluorescence images were taken using a color video camera (DP72, 12.8 megapixel CCD; Olympus), a microscope (IX50; Olympus) and the cellF software (version 3.3; Olympus). Non-probe incubations served as negative controls.

### RT-qPCR

RT-qPCR was performed for TMEV, *Isg15*, *Eif2ak2* (PKR), *Tnfa*, *Il1a*, *Il1b*, *Il4*, *Il6*, *Il10*, *Il12*, *Ifng*, *Tgfb1*, and three housekeeping genes (*Gapdh*, *Actb*, *Hprt1*) using the AriaMx Real-time PCR System (Agilent Technologies Deutschland GmbH) and Brilliant III Ultra-Fast SYBR^®^QPCR Master Mixes (Agilent Technologies Deutschland GmbH) (35).

### Statistical Analysis

Statistical analysis was performed using Prism 9 software (GraphPad). Survival and clinical data were analyzed using Mantle-Cox tests and Sidak multiple comparisons tests, respectively. Mann-Whitney tests were used to evaluate histological, immunohistochemical, virological and RT-qPCR data.

## Results

### IFN-β Deficiency Results in Rapid Death After TMEV-DA But Not TMEV-BeAn Infection

**Figure 1:**
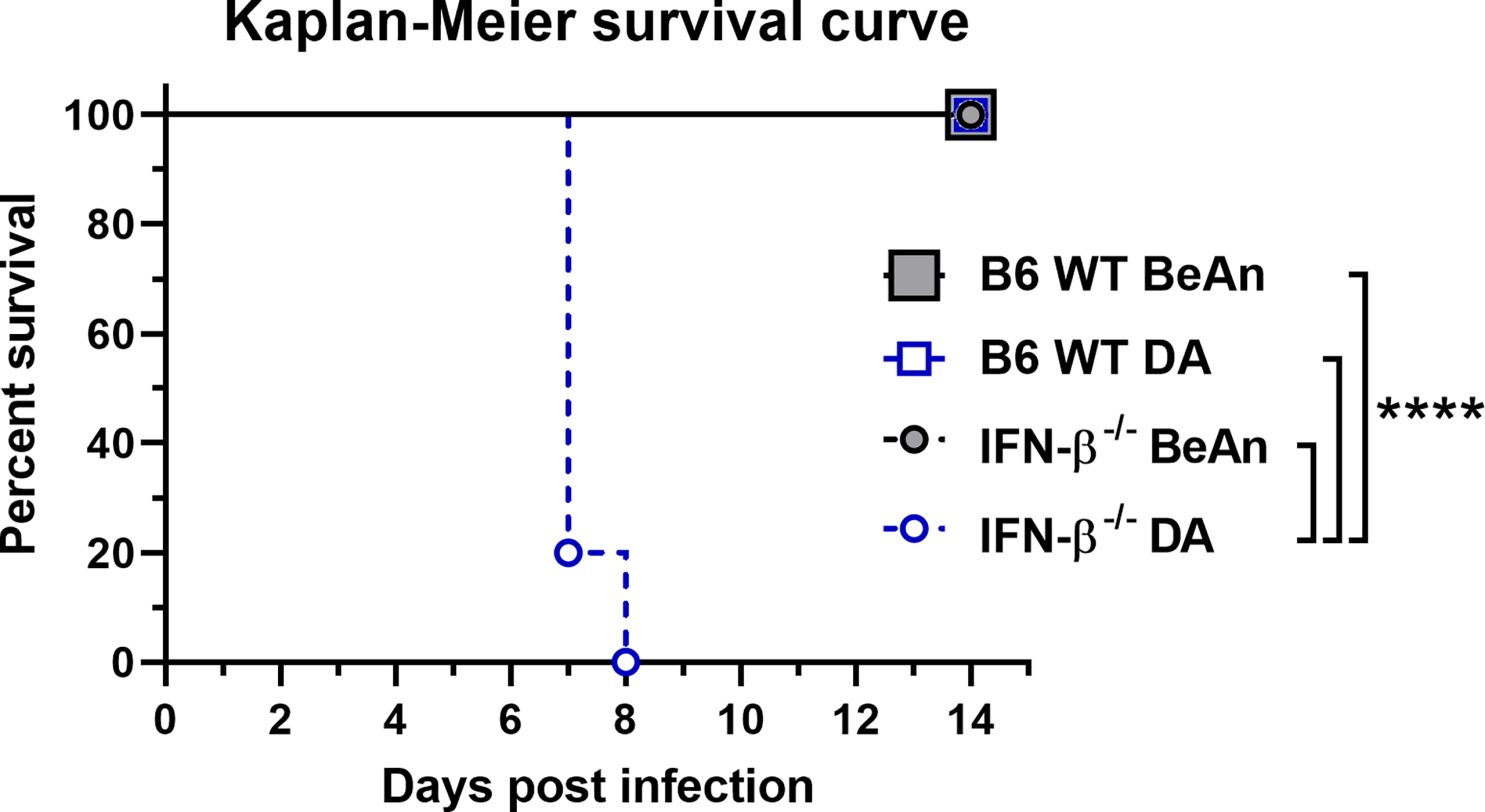
IFN-β is critical for the survival of *C57BL*/6 (B6) mice after infection with the DA but not the BeAn strain of TMEV. B6 *wild type* (WT) and IFN-β^-/-^ mice (n=10) were intracranially infected with 1 x 10^5^ PFU of TMEV-BeAn or TMEV-DA and survival was monitored twice a day. Mantle-Cox tests: ****p < 0.0001.

**Figure 2:**
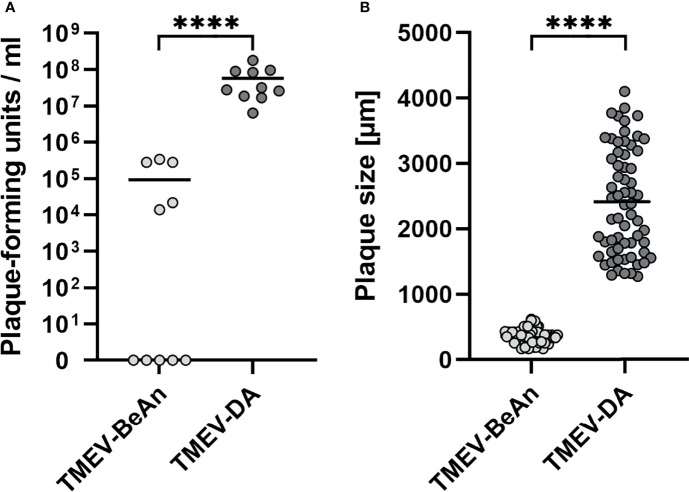
Low numbers of infectious viral particles were found in IFN-β^-/-^ mice after TMEV-BeAn infection, whereas TMEV-DA-infected IFN-β^-/- ^mice developed a high viral load and had to be euthanized due to severe clinical disease **(A)**. TMEV-BeAn and TMEV-DA induce small uniform plaques and large heterogeneous plaques, respectively **(B)**. IFN-β^-/-^ mice were intracranially infected with 1 x 10^5^ PFU of TMEV-BeAn or TMEV-DA. Plaque analysis was performed on L cells and used to detect infectious viral particles in the brain of TMEV-infected mice **(A)**. Moreover, plaque sizes of TMEV strains used for mouse infection were determined **(B)**. Mann-Whitney tests demonstrated statistically significant differences between the two TMEV strains (****p < 0.0001). Shown are all data points with means.

**Figure 3:**
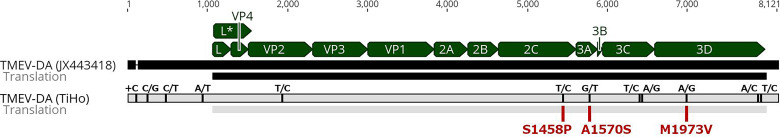
Sequencing revealed few differences between the TMEV-DA reference strain (JX443418) and the sequenced isolate (TiHo). Black, vertical lines in the upper track with the label directly above indicate 12 nucleotide differences. Red lines in the lower track with the label below indicate the amino acid changes.

Despite the resistance of B6 *wild type* (WT) mice to TMEV infection, 80% (8/10) of DA-infected IFN-β^-/-^ mice had to be killed at 7 *days post infection* (dpi) and another 20% (2/10) at 8 dpi due to severe clinical disease, whereas all BeAn-infected mice (10/10) survived (**Figure 1**). Lethal disease in DA-infected IFN-β^-/-^ mice was associated with a high viral load, which was revealed by plaque assay (**Figure 2A**) and immunohistochemistry (**Supplementary Figure 1**). In contrast, low numbers of infectious viral particles were found in surviving BeAn-infected IFN-β^-/-^ mice at 14 dpi (**Figure 2A**). Interestingly, plaque analysis showed that BeAn induced small uniform plaques, whereas DA infection results in larger heterogeneous plaques (**Figure 2B** and **Supplemental Figure 2**). This prominent difference in plaque size was found in TMEV strains used for intracerebral infection and viruses re-isolated from brain tissue of infected animals (**Supplementary Figure 3**). The unanticipated difference in virulence instigated a detailed analysis of TMEV strains used for animal experiments and their interaction with IFN-β signaling. Virus sequencing detected 12 nucleotide differences in the TMEV-DA genome compared to a reference sequence (JX443418), but these changes only caused three amino acid changes in the virus proteins 2C, 3A and 3D, respectively (**Figure 3** and **Supplemental Table 1**). Moreover, a phylogenetic analysis demonstrated that TMEV strains used in the present study are highly similar to TMEV strains used by other research groups (**Supplemental Figure 4**).

In summary, DA but not BeAn induces a lethal disease in IFN-β^-/-^ mice. These virus strains also differ in their *in vitro* growth characteristics.

### Luciferase Reporter Mice Show a Lower IFN-β Expression After a TMEV-DA Compared to a TMEV-BeAn Infection

**Figure 4:**
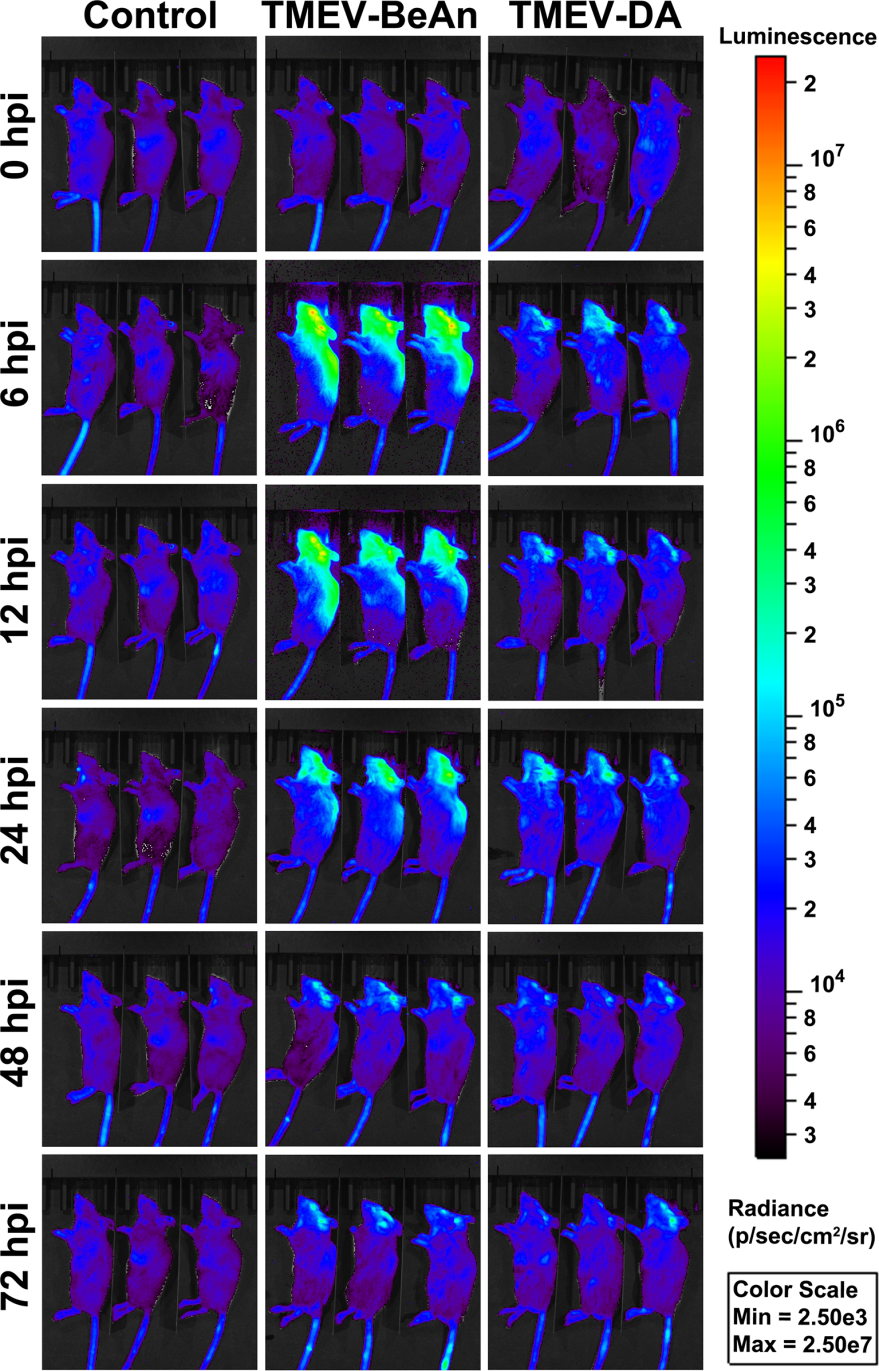
Strong IFN-β expression in C57BL/6 mice infected with TMEV-BeAn but not TMEV-DA. Groups of three IFN-β luciferase reporter mice (IFN-β^Δβ-luc/wt^) received an intracranial injection of cell culture medium (Dulbecco’s Modified Eagle Medium) as control or were intracranially infected with 1 x 10^5^ PFU of TMEV-BeAn or TMEV-DA, respectively. For analysis of luciferase expression, luciferin was i.v. injected at 0, 6, 12, 24, 48 and 72 hours post infection (hpi), and mice were monitored in an IVIS Spectrum CT *in vivo* imager.

*In vivo* imaging using luciferase reporter mice demonstrated a strong IFN-β expression already 6 hours after BeAn infection, whereas mice infected with DA only showed a limited IFN-β expression at this time point. Nevertheless, the strong IFN-β expression after BeAn infection rapidly declined and was similar to DA-infected mice at 3 dpi (**Figure 4**). IFN-β mRNA expression at 1 dpi was localized to the CA1 area of the hippocampus and ependymal cells of the third ventricle using FISH (**Supplemental Figure 5**).

In summary, IFN-β expression is restricted in DA- compared to BeAn-infected mice, which produce IFN-β mRNA in the hippocampus and third ventricle.

### IFN-β^-/-^ Mice Show Increased Inflammation, Viral Load and Astrogliosis After TMEV-BeAn Infection due to Lacking IFN-β Expression of Non-Neuroectodermal Cells

**Figure 5:**
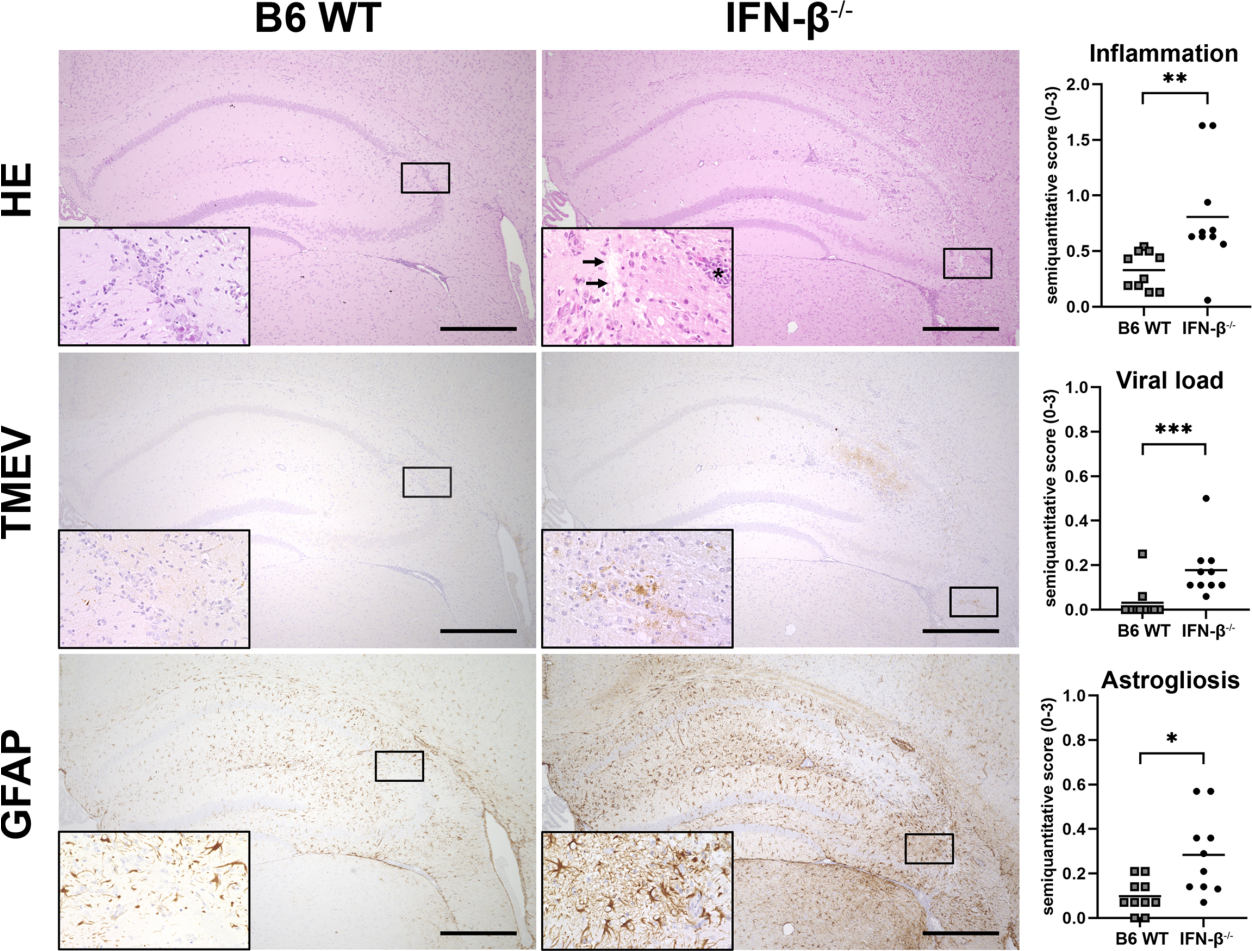
IFN-β restricts inflammatory cell infiltration, viral replication and astrogliosis. *C57BL*/6 *wild type* (B6 WT) and IFN-β^-/-^ mice were intracranially infected with 1 x 10^5^ PFU of TMEV-BeAn. Inflammation, viral load and astrogliosis was evaluated semiquantitatively in the cerebrum at 14 days post infection. Upper row: Enhanced perivascular mononuclear infiltrates (asterisk) as well as edema and neuronal loss (arrows) in the hippocampus of IFN-β^-/-^ compared to WT mice. *Hematoxylin and eosin* (HE) stain. Middle row: The lesion in IFN-β^-/-^ mice contains a higher amount of TMEV antigen compared to B6 WT mice. *Immunohistochemistry* (IHC) using the *avidin-biotin complex* (ABC) method. Lower row: Severe astrogliosis in the hippocampus of IFN-β^-/-^ but not WT mice revealed by *glial fibrillary acidic protein* (GFAP) staining. IHC using the ABC method. Bars = 500 µm. Insets show higher magnifications. Mann-Whitney tests revealed higher inflammation (**p = 0.0013), viral load (***p = 0.0007), and astrogliosis (*p = 0.013) in IFN-β^-/-^ compared to B6 WT mice after TMEV-BeAn infection. Shown are all data points with means.

**Figure 6:**
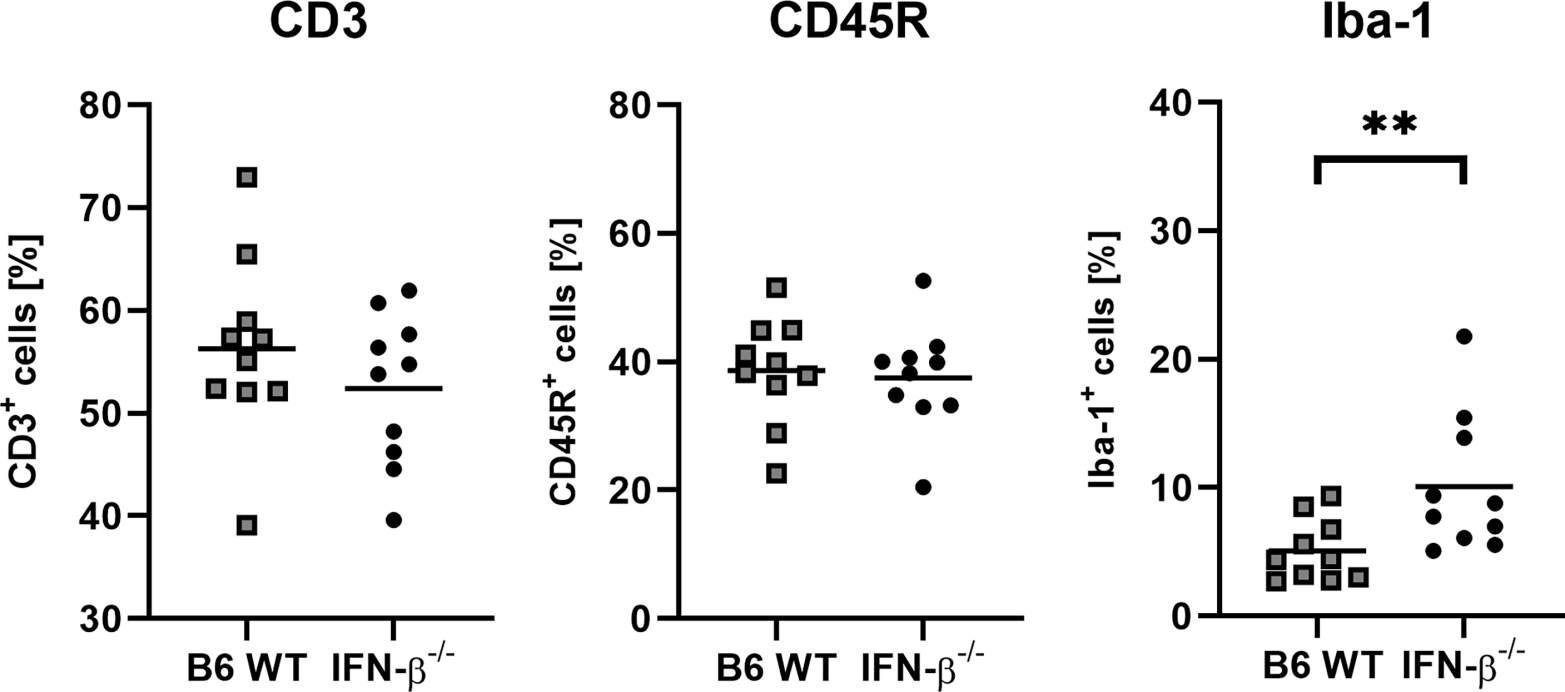
IFN-β^-/-^ mice show slightly increased percentages of perivascular Iba-1^+^ macrophages compared of *C57BL/6 wild type* (B6 WT) mice in the cerebrum at 14 days after TMEV-BeAn infection. Percentages of perivascular CD3^+^ T cells and CD45R^+^ B cells are not affected by IFN-β^-/-^ deficiency. Mann-Whitney test: (**p = 0.0089). Shown are all data points with means.

**Figure 7:**
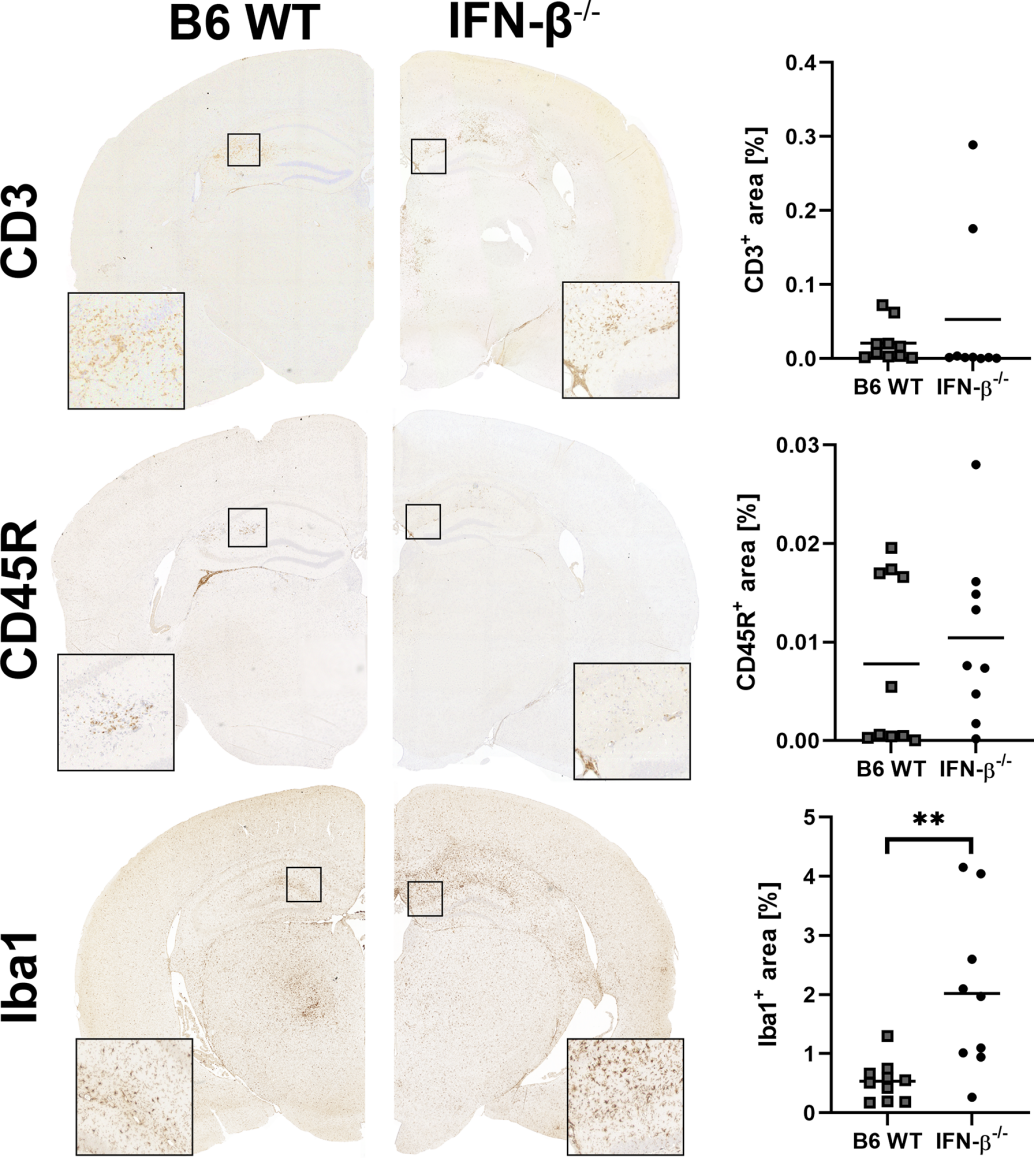
IFN-β^-/-^ mice show an increased number of Iba-1^+^ microglia/macrophages compared of *C57BL/6 wild type* (B6 WT) mice in the brain at 14 days after TMEV-BeAn infection. In contrast, IFN-β^-/-^ deficiency did not change the infiltration of lymphocytes (CD3^+^ T cells and CD45R^+^ B cells). Immunohistochemistry (IHC) using the avidin-biotin complex (ABC) method. Percentages of immunopositive area were determined using morphometry of immunohistochemically stained brain sections (cerebrum with hippocampus, thalamus and hypothalamus; cerebellum with medulla oblongata). Mann-Whitney test: (**p=0.0030). Shown are all data points with means.

BeAn-infected IFN-β^-/-^ mice showed higher numbers of perivascular inflammatory infiltrates, more viral antigen and an increased astrogliosis in the cerebrum at 14 dpi compared to B6 WT mice (**Figure 5**). Moreover, the percentage of perivascular Iba-1^+^ macrophages was higher in IFN-β^-/-^ than in B6 WT mice, whereas no difference was found in the percentages of perivascular CD3^+^ T cells and CD45R^+^ B cells (**Figure 6**). Likewise, a quantification of Iba-1^+^, CD3^+^ and CD45R^+^ cells in the brain demonstrated that IFN-β^-/-^ mice display a higher number of intraparenchymal microglia/macrophages but a similar infiltration of lymphocytes after TMEV infection (**Figure 7** and **Supplemental Figure 6**). Hippocampal cell loss was similar in IFN-β^-/-^ and B6 WT mice (**Supplemental Figure 7**).

**Figure 8:**
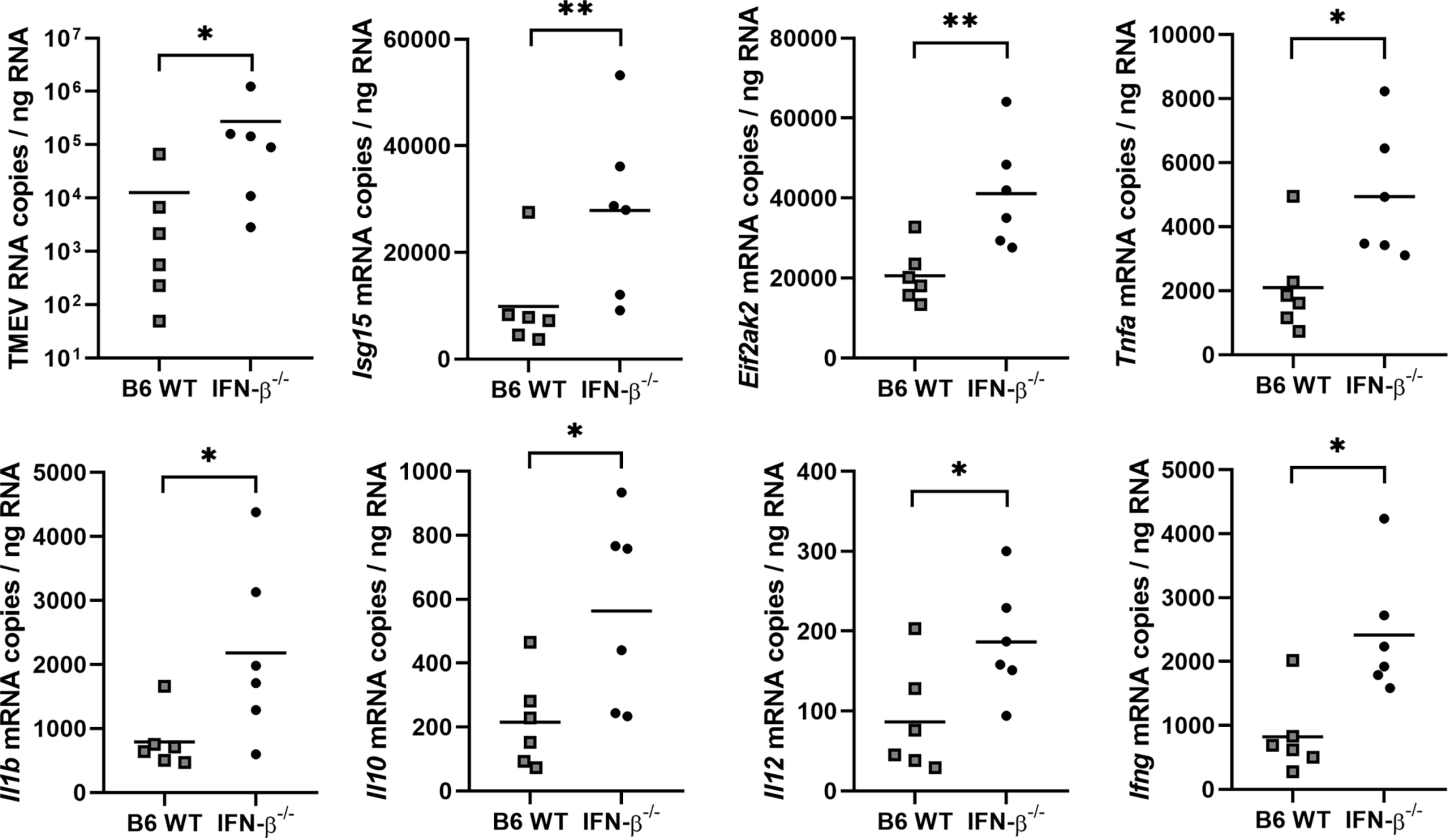
IFN-β restricts viral replication and modulates the cytokine milieu in the cerebrum at 14 days after TMEV-BeAn infection. *C57BL/6 wild type* (B6 WT) and IFN-β^-/-^ mice were intracranially infected with 1 x 10^5^ PFU of TMEV-BeAn. Mann-Whitney tests of RT-qPCR data demonstrated higher levels of TMEV RNA (*p=0.0152) as well as *Isg15* (**p=0.0087), *Eif2ak2* (PKR) (**p=0.0087), *Tnfa* (*p=0.026), *Il1b* (*p=0.0152), *Il10* (*p=0.0411), *Il12* (*p=0.0411) and *Ifng* (*p=0.0152) mRNA in IFN-β^-/-^ compared WT mice. Shown are all data points with means.

*Reverse transcription-quantitative polymerase chain reaction* (RT-qPCR) revealed higher levels of TMEV RNA and *Eif2ak2* (PKR), *Isg15*, *Tnfa*, *Il1b*, *Il10*, *Il12* and *Ifng* mRNA in the cerebrum of IFN-β^-/-^ compared to B6 WT mice at 14 dpi (**Figure 8**). No difference in *Il4*, *Il6* and *Tgfb1* mRNA transcripts was detected in the cerebrum between these mice at 14 dpi and for all genes at 98 dpi (**Supplemental Figure 8**).

**Figure 9:**
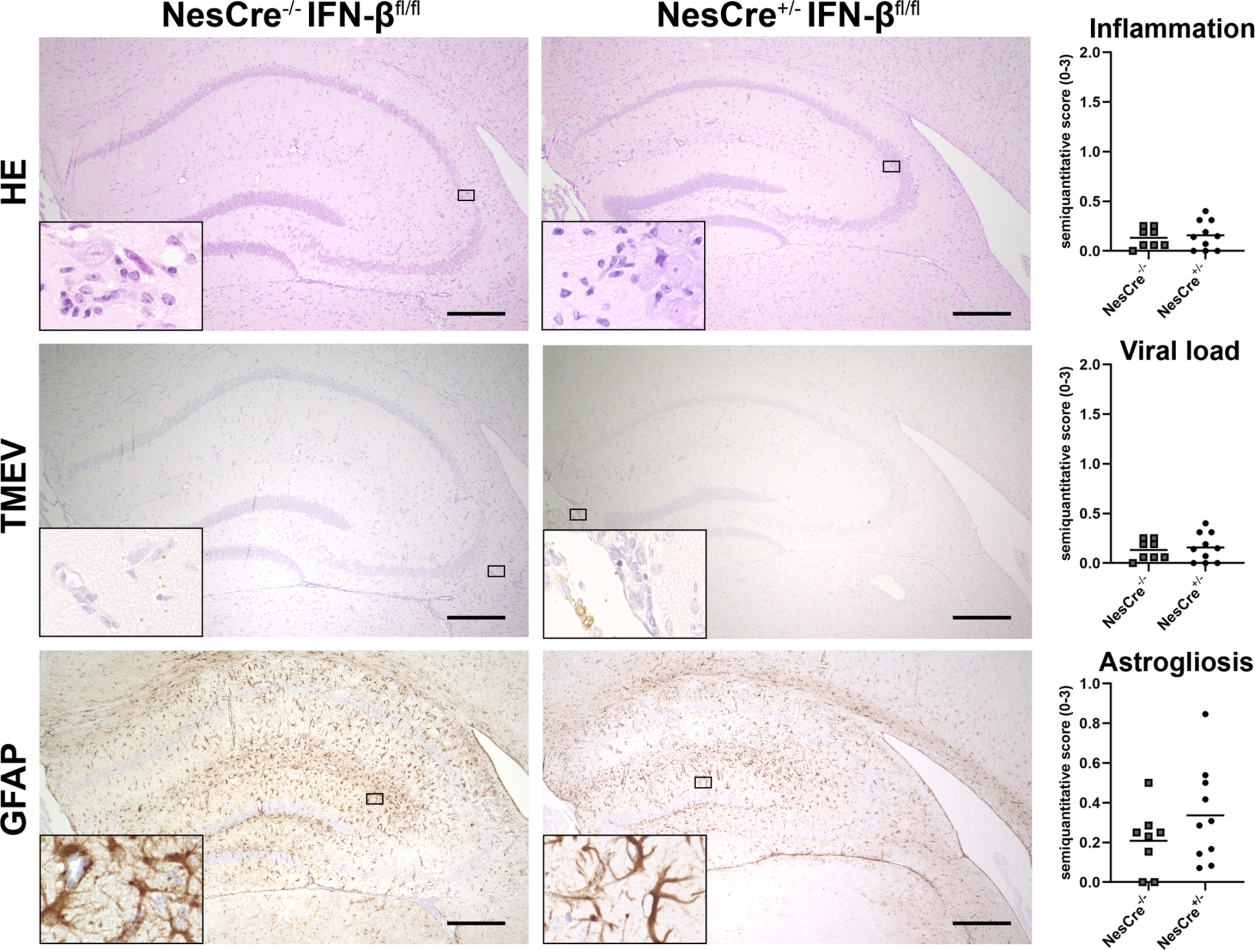
NesCre^+/-^ IFN-β^fl/fl^ mice do not develop more severe lesions than wild type littermates after TMEV-BeAn infection. NesCre^-/-^ IFN-β^fl/fl^ and NesCre^+/-^ IFN-β^fl/fl^ mice were intracranially infected with 1 x 10^5^ PFU of TMEV-BeAn. Inflammation, viral load and astrogliosis was evaluated semiquantitatively in the cerebrum at 14 days post infection. Upper row: Perivascular mononuclear infiltrates are similar in the cell-type specific knockout mice and wild type littermates. *Hematoxylin and eosin* (HE) stain. Middle row: Very low amount of TMEV antigen in both mouse strains. *Immunohistochemistry* (IHC) using the *avidin-biotin complex* (ABC) method. Lower row: An astrogliosis is present in the hippocampus of both mouse strains revealed by *glial fibrillary acidic protein* (GFAP) staining. IHC using the ABC method. Bars = 150 µm. Insets show higher magnifications. Mann-Whitney tests did not detect statistically significant differences between cell-type specific knockout mice and wild type littermates. Shown are all data points with means.

To investigate the relevance of central and peripheral IFN-β expression, NesCre^+/-^ IFN-β^fl/fl^ mice, which do not express IFN-β in neuroectodermal cells (neurons, astrocytes and oligodendrocytes), and wild type littermates were intracranially infected with TMEV-BeAn. Interestingly, lack of IFN-β expression in neuroectodermal cells did not result in enhanced inflammation, viral load, astrogliosis and hippocampal cell loss (**Figure 9** and **Supplemental Figure 7**). Consequently, inhibition of viral replication and efficient immune responses seem to be dependent on IFN-β expression of non-neuroectodermal cells after BeAn infection.

### TMEV-BeAn Persists in IFN-β^-/-^ Mice and Induced a Mild Demyelination in the Spinal Cord White Matter

**Figure 10:**
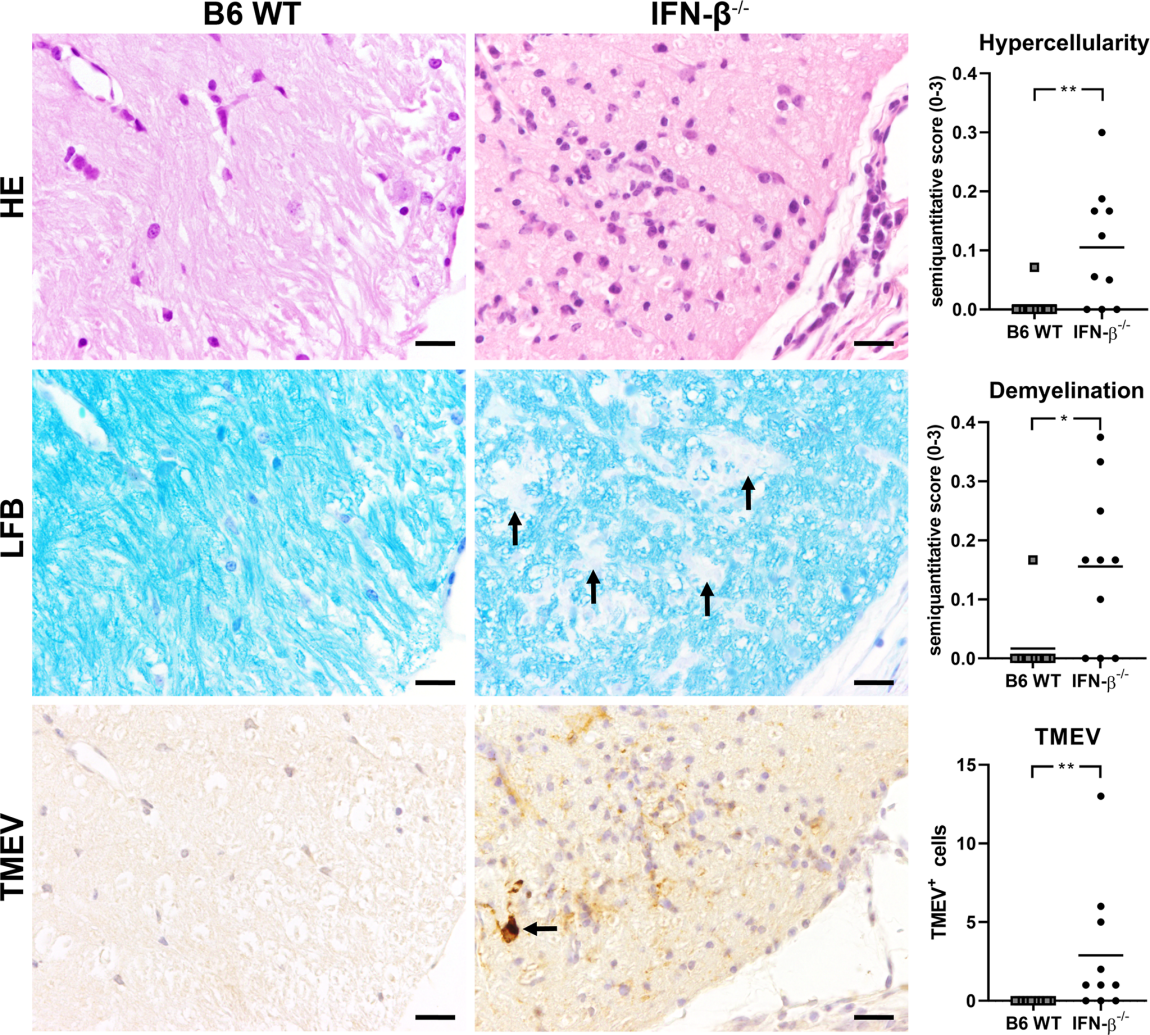
IFN-β deficiency results in hypercellularity, mild demyelination and virus persistence in the spinal cord of *C57BL/6* (B6) mice at 98 days after TMEV-BeAn infection. Upper row: histological lesions in the spinal cord of B6 *wild type* (WT) and IFN-β^-/-^ mice. Note increased cellularity in the white matter and multifocal mononuclear infiltrates in the meninges of IFN-β^-/-^ mice. *Hematoxylin and eosin* (HE) stain. Middle row: Mild demyelination in the white matter of the spinal cord of IFN-β^-/-^ mice (arrows). *Luxol fast blue stain* (LFB). Lower row: immunohistochemical detection of TMEV antigen. Note virus-infected cells in the spinal cord white matter (arrow). Avidin-biotin complex immunoperoxidase method. Bars = 20 µm. Mann-Whitney tests demonstrated significantly increased hypercellularity (**p=0.0079), demyelination (*p=0.0126), and TMEV-infected cells (**p=0.0031) in the spinal cord of IFN-β^-/-^ compared to B6 WT mice at 98 dpi. Shown are all data points with means.

**Figure 11:**
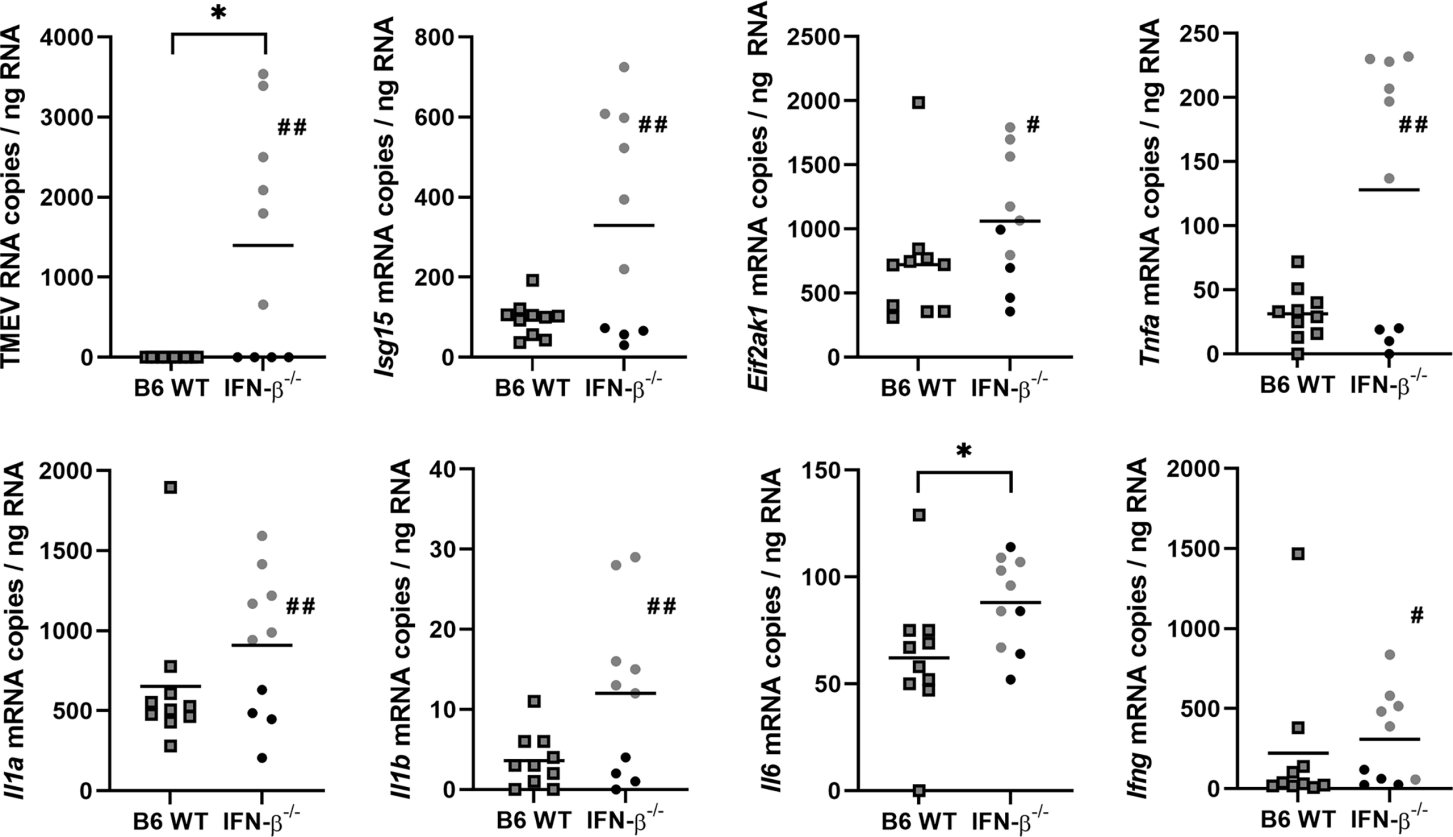
Virus persistence results in increased expression of *interferon stimulated genes* (ISGs) and pro-inflammatory cytokines in the spinal cord of IFN-β^-/-^ mice at 98 days after TMEV-BeAn infection. *C57BL*/6 *wild type* (B6 WT) and IFN-β^-/-^ mice were intracerebrally infected with 1 x 10^5^ PFU of TMEV-BeAn. Mann-Whitney tests of RT-qPCR data demonstrated higher levels of TMEV RNA (*p=0.0108) and *Il6* (*p=0.0408) mRNA in the spinal cord of IFN-β^-/-^ compared to WT mice. Note that TMEV RNA was detected in six IFN-β^-/-^ mice (gray dots), whereas four IFN-β^-/-^ mice (black dots) eliminated the virus. Moreover, Mann-Whitney tests revealed higher levels of TMEV RNA as well as *Isg15*, *Eif2ak1*, *Tnfa*, *Il1a*, *Il1b* and *Ifng* mRNA in TMEV^+^ compared to TMEV^-^ IFN-β^-/-^ mice (^#^p < 0.05; ^##^p < 0.01). Shown are all data points with means.

After BeAn infection, IFN-β^-/-^ and WT mice did not differ in their clinical scores, weight and RotaRod performance up to 98 dpi (**Supplemental Figure 9**). In addition, histology did not detect morphological lesions in the brain of infected IFN-β^-/-^ and B6 WT mice at 98 dpi. However, TMEV persisted in the spinal cord of IFN-β^-/-^ mice at a low level and caused an increased cellularity in the spinal cord white matter and a mild demyelination at 98 dpi (**Figure 10**). Correspondingly, RT-qPCR detected TMEV RNA in the spinal cord of six out of ten IFN-β^-/-^ mice but not in B6 WT mice at 98 dpi, which was associated with higher *Il6* mRNA levels in IFN-β^-/-^ compared to B6 WT mice. Finally, IFN-β^-/-^ mice persistently infected with TMEV had higher *Isg15*, *Eif2ak1*, *Tnfa*, *Il1a*, *Il1b* and *Ifng* mRNA levels in the spinal cord at 98 dpi compared to IFN-β^-/-^ mice without TMEV (**Figure 11**).

In summary, IFN-β^-/-^ mice showed a severely restricted capacity to eliminate BeAn from the CNS leading to ongoing inflammation and mild demyelination in the spinal cord despite of higher ISG expression.

## Discussion

The present study aimed to investigate the role of IFN-β during TMEV infection. For this purpose IFN-β^-/-^ mice were generated on a C57BL/6 background that is usually resistant to TMEV-IDD (1). Surprisingly, all IFN-β^-/-^ mice died one week after infection with the DA strain of TMEV, whereas the TMEV-BeAn strain did not cause death in these mice. TMEV strains and substrains are known to differ in their capacity to cause brain inflammation and neurodegeneration and can even cause early seizures and epilepsy in C57BL/6 mice (2, 29). A comparison between the sequences of two TMEV-BeAn substrains detected four coding changes located within the VP1 and VP3 (capsid), 3A (nonstructural protein) and 3D (polymerase) genes, which most likely contribute to these differences in neurovirulence (29). Likewise, poliovirus protein 3A inhibits the transport from the endoplasmic reticulum to the Golgi apparatus, blocks TNF-induced apoptosis and even limits IL-6, IL-8 and IFN-β secretion during viral infections (36–38). The present TMEV-DA strain exhibited three coding changes in the virus proteins 2C, 3A and 3D compared to a reference sequence (JX443418), which might also affect its virulence. Mutations in the N-terminus of the nucleosid triphosphatase (NTPase) 2C can impair virus replication but the detected amino acid substitution (S1458P) was located in the C-terminus of this virus protein making functional relevance unlikely (39, 40). Interestingly, our BeAn and DA strains produced small (< 1 mm) and larger heterogeneous plaques (1-5 mm), respectively. Highly neurovirulent TMEV strains such as GDVII and FA are known to induce large plaques in BHK-21 cells, whereas low neurovirulent strains including DA, TO, WW, Yale and BeAn produce small plaques (41, 42). When assayed on L2 cells, small (0.75 ± 0.13 mm) and large (1.51 ± 0.16 mm) plaque forming variants of the DA strain were described (43). Nevertheless, the small plaque forming variant caused severe lesions in SJL/J and C57BL/6 mice, whereas the large plaque forming variant was attenuated most likely due to mutated L and L* proteins (43, 44). In contrast, TMEV strains used in the present study did not have coding changes in these proteins, which are known to interfere with IFN-I gene transcription and the antiviral OAS/RNase L pathway (8–13). Nonetheless, luciferase reporter mice demonstrated a restricted IFN-β expression in DA- compared to BeAn-infected mice indicating a disturbed IFN-I signaling. The higher virulence of the DA compared to the BeAn strain is also related to a restricted avidity and epitope recognition of CD8^+^ T cells (3, 45, 46). Consequently, DA seems to block early innate immune responses and prevent detection by virus-specific T cells more efficiently than BeAn resulting in fatal disease in IFN-β^-/-^ mice.

Another major finding of the present study was that BeAn-infected IFN-β^-/-^ mice showed higher inflammation and viral load as well as stronger astrogliosis than WT mice. IFN-β deficiency did not modify lymphocyte infiltration into the brain at 14 dpi but might have affected their activation status and antiviral activity. IFN-β controls T cell activation, pro- and anti-inflammatory cytokine secretion and blood-brain barrier integrity during neuroinflammatory diseases (47, 48). IFN-β can either promote or inhibit T cell activation, proliferation, differentiation and survival, which primarily depends on the timing of IFN-I exposure and T cell receptor stimulation (49). However, the TMEV-BeAn strain persisted in IFN-β^-/-^ mice resulting in ongoing inflammatory processes in the spinal cord white matter and even mild demyelination at 98 dpi. The genetic background of C57BL/6 mice can influence the extent of clinical disease, neuroinflammation and neurodegeneration after TMEV-BeAn infection (2). Nonetheless, differences in the genetic background of IFN-β^-/-^ and WT mice cannot explain virus persistence and demyelinating lesions in IFN-β^-/-^ mice, because both 129 mice (original strain of IFN-β^-/-^ mice) and C57BL/6 mice including all substrains are highly resistant to TMEV-IDD and eliminate the virus in the acute phase of the disease (1, 50). Interestingly, IFN-β^-/-^ mice also developed more extensive inflammation and demyelination in *experimental autoimmune encephalomyelitis* (EAE), an animal model of human *multiple sclerosis* (MS), underlining the prominent role of IFN-β in the prevention of demyelinating diseases (51). EAE lesions in IFN-β^-/-^ mice were not caused by increased T cell priming or encephalitogenicity of T cells nor a skewed balance between Th1 and Th2 responses. In contrast, locally produced IFN-β seems to limit microglia activation and TNF-α production. This inhibitory effect on resident antigen presenting cells might reduce the *in situ* T cell reactivation and their cytokine production (51). In general, IFNAR signaling of myeloid cells such as microglia/macrophages but not of T cells or neurons seem to mediate the protective effects of IFN-β during autoimmune CNS inflammation (52, 53).

Similar to the results of the above-described EAE study, TMEV-infected IFN-β^-/-^ mice developed an increased expression of the pro-inflammatory cytokine TNF-α, which can be produced by microglia, astrocytes and infiltrating immune cells (35). Likewise, both resident glial and infiltrating immune cells can contribute to increased expression of IL-1β, IL-10, IL-12 and IFN-γ in the cerebrum of IFN-β^-/-^ compared to B6 WT mice at 14 dpi. High TNF-α, IL-1β, IL-10 and IFN-γ levels might also have caused astrogliosis in TMEV-infected IFN-β^-/-^ mice (54–56), which can be a self-reinforcing process, because reactive astrocytes can produce themselves various chemokines and cytokines to regulate CNS inflammation (35, 57). IL-12 and IFN-γ are pro-inflammatory Th1 cytokines and support protective *cytotoxic T cell* (CTL) responses, which are critical for resistance to TMEV-IDD (6, 58–60). Nonetheless, elevated expression of the anti-inflammatory cytokine IL-10 inhibits Th1 responses critical for TMEV elimination (7). Moreover, high-level expression of IL-1β can elevate pathogenic Th17 responses, which facilitate viral persistence and inhibit T cell cytotoxicity (61, 62). However, increased cytokine expression might simply be the result and not the cause of enhanced viral replication in TMEV-infected IFN-β^-/-^ mice limiting conclusions about its pathogenic role. A recent VSV study revealed that IFN-I stimulation of neurons and astrocytes is critical for full microglia activation and restriction of viral spread (63). Similarly, BeAn-infected IFNAR^-/-^ mice developed rapid fatal encephalitis due to less efficient stimulation of virus-specific T cells by antigen-presenting cells and hence unrestricted viral replication (16). This indirect effect of IFN-I on microglia activation strikingly contrasts the direct inhibitory effects of IFNAR signaling on CNS myeloid cells described in EAE. Consequently, the absence of IFN-β stimulation of neurons and astrocytes might impair antigen presentation in the present BeAn-infected IFN-β^-/-^ mice and thereby efficient antiviral immune responses despite of increased ISG expression.

IFN-I can be produced by basically all types of nucleated cells, but plasmacytoid dendritic cells are main producers of IFN-I during viral infections (64). In the brain, abortively infected astrocytes are the major source of IFN-β after infection with different neurotropic viruses including TMEV, VSV and rabies virus. Moreover, microglia/macrophages and to a minor extent neurons contribute to local IFN-β expression (20, 65). To investigate the importance of IFN-β expression by resident CNS cells on the outcome of TMEV infection, we used NesCre^+/-^ IFN-β^fl/fl^ mice, which do not express IFN-β in neuroectodermal cells. These cell-type specific knockout mice did not develop more severe brain lesions than wild type littermates demonstrating that the resistance of C57BL/6 mice to TMEV-IDD does not depend on IFN-β expression of neurons, astrocytes and oligodendrocytes. In contrast, IFN-β production of microglia and peripheral immune cells as well as the expression of other IFN-I family members might prevent severe disease in BeAn-infected NesCre^+/-^ IFN-β^fl/fl^ mice.

In conclusion, IFN-β deficiency in C57BL/6 mice results in fatal disease after DA infection or virus persistence and demyelinating disease after BeAn infection. These results demonstrate a critical function of this innate cytokine for effective antiviral immune responses and prevention of demyelination processes. Moreover, lack of severe disease in BeAn-infected NesCre^+/-^ IFN-β^fl/fl^ mice shows that the resistance of C57BL/6 mice to TMEV-IDD does not depend on IFN-β expression of neurons, astrocytes and oligodendrocytes. Further studies investigating the interactions of specific IFN-I pathway members with resident CNS and invading immune cells during virus-induced inflammatory processes of the brain and spinal cord will certainly help to understand the exact role of IFN-I in the development of long-term consequences of viral encephalitis.

## Data Availability Statement

The original contributions presented in the study are included in the article/**Supplementary Material**. Further inquiries can be directed to the corresponding author.

## Ethics Statement

The animal study was reviewed and approved by Niedersächsisches Landesamt für Verbraucherschutz und Lebenmittelsicherheit, Oldenburg, Germany, permission number: 33.12-42502-04-14/1656.

## Author Contributions

MB – drafting and manuscript preparation, data collection and data analysis. SR, DL, JG, CD, VN, and MS – data collection and data analysis. UK – idea generation and manuscript editing. AB, TS, and WB – manuscript editing. IG – idea generation, drafting and manuscript preparation, data analysis and manuscript editing. All authors contributed to the article and approved the submitted version.

## Funding

This work was supported by the Niedersachsen-Research Network on Neuroinfectiology (N-RENNT) of the Ministry of Science and Culture of Lower Saxony to WB and UK. DL was funded by the China Scholarship Council (File No. 01606170128). This Open Access publication was funded by the Deutsche Forschungsgemeinschaft (DFG, German Research Foundation) within the programme LE 824/10-1 "Open Access Publication Costs" and University of Veterinary Medicine Hannover, Foundation.

## Conflict of Interest

CD was and UK is employed by Twincore, Centre for Experimental and Clinical Infection Research, a joint venture between the Hannover Medical School and the Helmholtz Centre for Infection Research.

The remaining authors declare that the research was conducted in the absence of any commercial or financial relationships that could be construed as a potential conflict of interest.

## Publisher’s Note

All claims expressed in this article are solely those of the authors and do not necessarily represent those of their affiliated organizations, or those of the publisher, the editors and the reviewers. Any product that may be evaluated in this article, or claim that may be made by its manufacturer, is not guaranteed or endorsed by the publisher.
