# Supplementary material for: IFN-β Deficiency Results in Fatal or Demyelinating Disease in C57BL/6 Mice Infected With Theiler’s Murine Encephalomyelitis Viruses
Source: Front Immunol. 2022 Feb 9;13:786940. doi: 10.3389/fimmu.2022.786940 (PMC8864290; doi:10.3389/fimmu.2022.786940)
Supplement: Supplementary file 1 [file DataSheet_1.pdf]

Supplementary Material

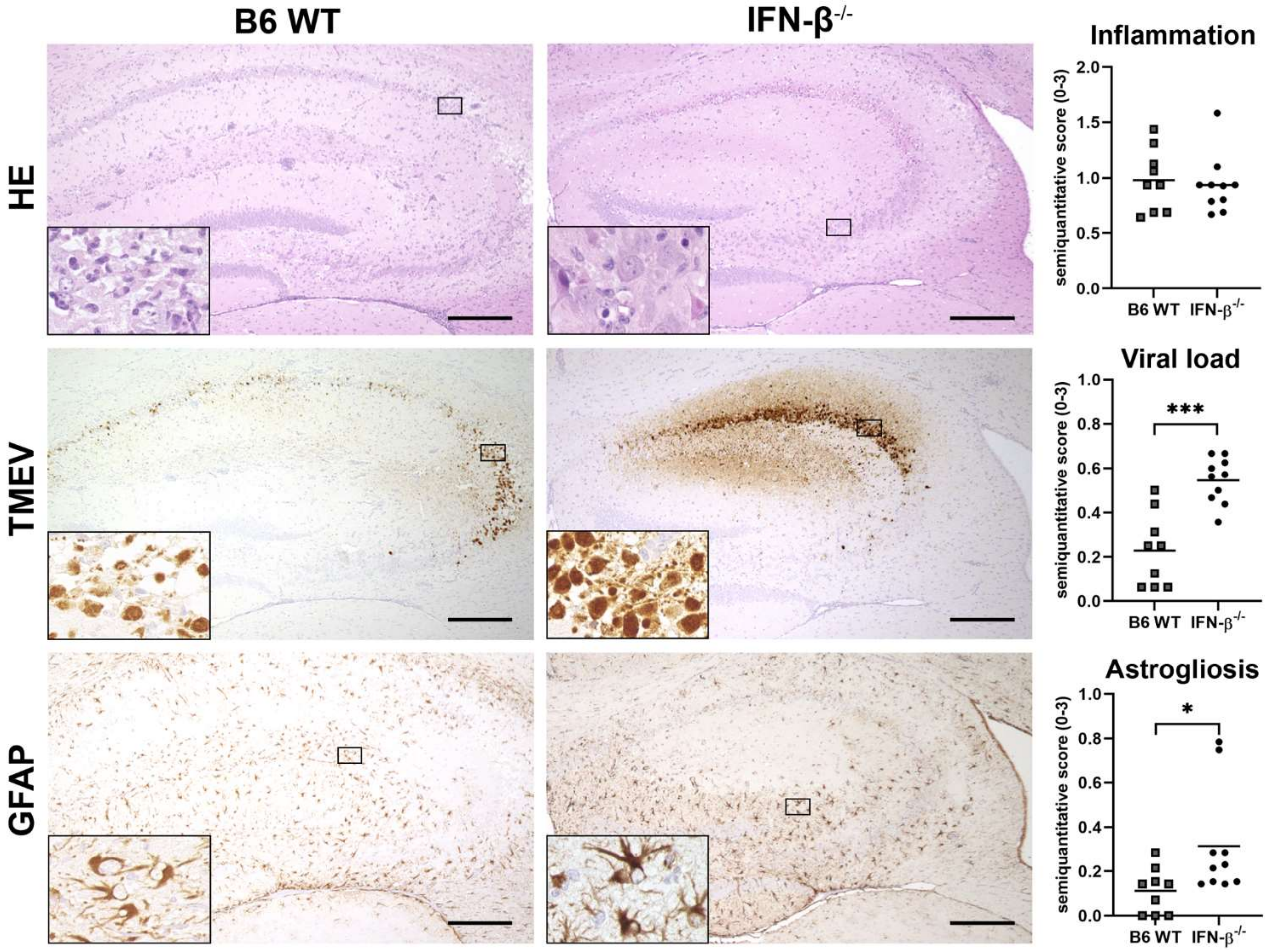

Supplemental Figure 1. *C57BL*/6 *wild type* (B6 WT) and IFN-$\beta^{-/-}$ mice were intracranially infected with 1 x 10^5 PFU of TMEV-DA. Inflammation, viral load and astrogliosis was evaluated semiquantitatively in the cerebrum at 7/8 days post infection. Upper row: Perivascular mononuclear infiltrates are similar in IFN-$\beta^{-/-}$ and WT mice. *Hematoxylin and eosin* (HE) stain. Middle row: The lesion in IFN-$\beta^{-/-}$ mice contains a higher amount of TMEV antigen compared to B6 WT mice. *Immunohistochemistry* (IHC) using the *avidin-biotin complex* (ABC) method. Lower row: Increased astrogliosis in the hippocampus of IFN-$\beta^{-/-}$ compared to WT mice revealed by *glial fibrillary acidic protein* (GFAP) staining. IHC using the ABC method. Bars = 250 µm. Insets show higher magnifications. Mann-Whitney tests revealed higher viral load (*** p=0.0004), and astrogliosis (* p=0.0408) in IFN-$\beta^{-/-}$ compared to B6 WT mice after TMEV-DA infection. Shown are all data points with means.

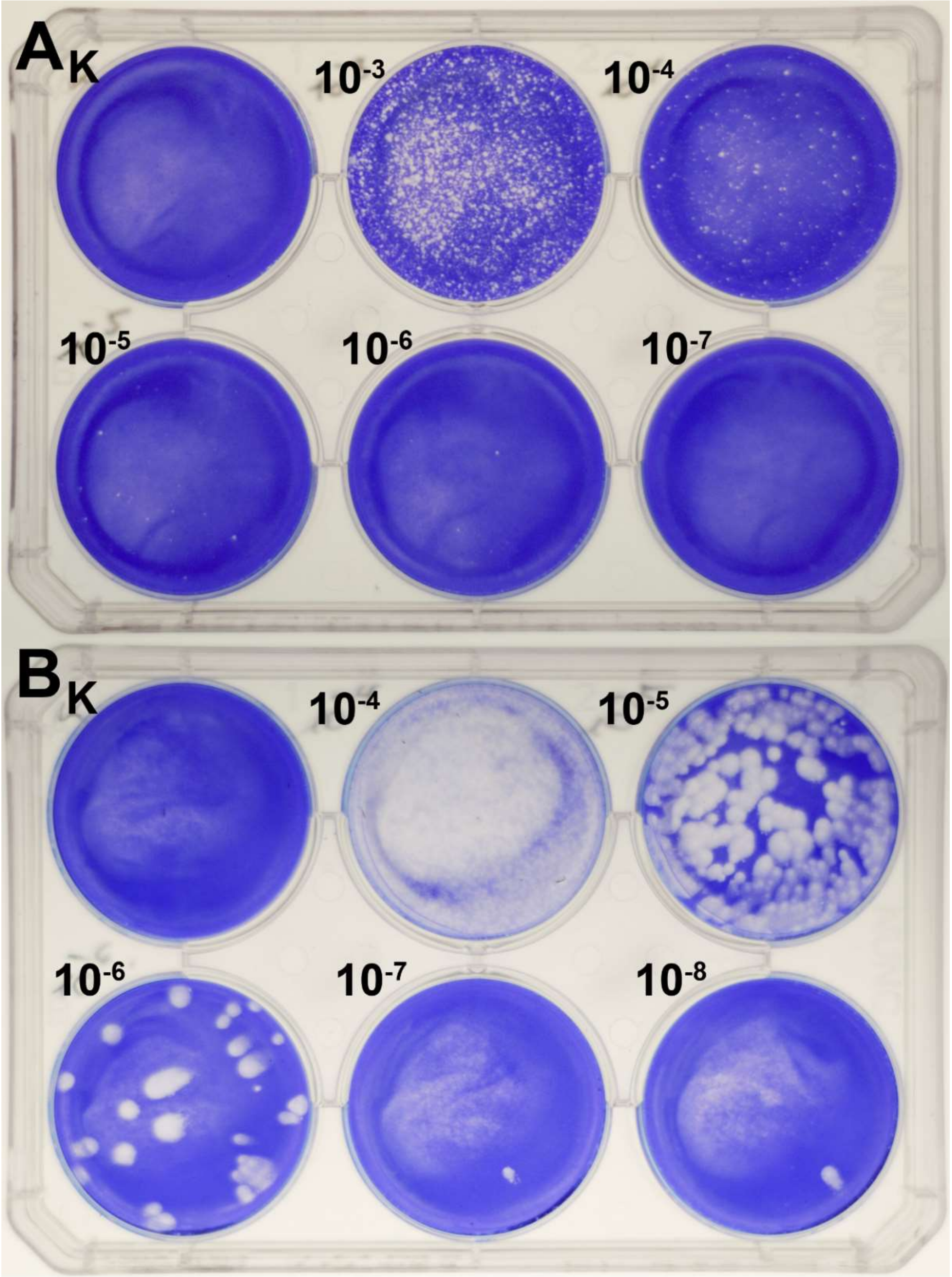

Supplemental Figure 2. Plaque assay of TMEV-BeAn (**A**) and TMEV-DA (**B**) strains used in the present study demonstrating uniform small plaques (**A**) or larger heterogeneous plaques (**B**), respectively. Serial dilutions of brain homogenates of cerebral tissue (10^{-3} to 10^{-7} or 10^{-4} to 10^{-8}) were added to 6-well culture plates of confluent L cells for 1 hour at room temperature. Subsequently, cells were covered with methyl cellulose. Three days later, cells were fixed with 10% buffered formalin and stained with crystal violet. K = control (incubation with Dulbecco's Modified Eagle Medium, DMEM).

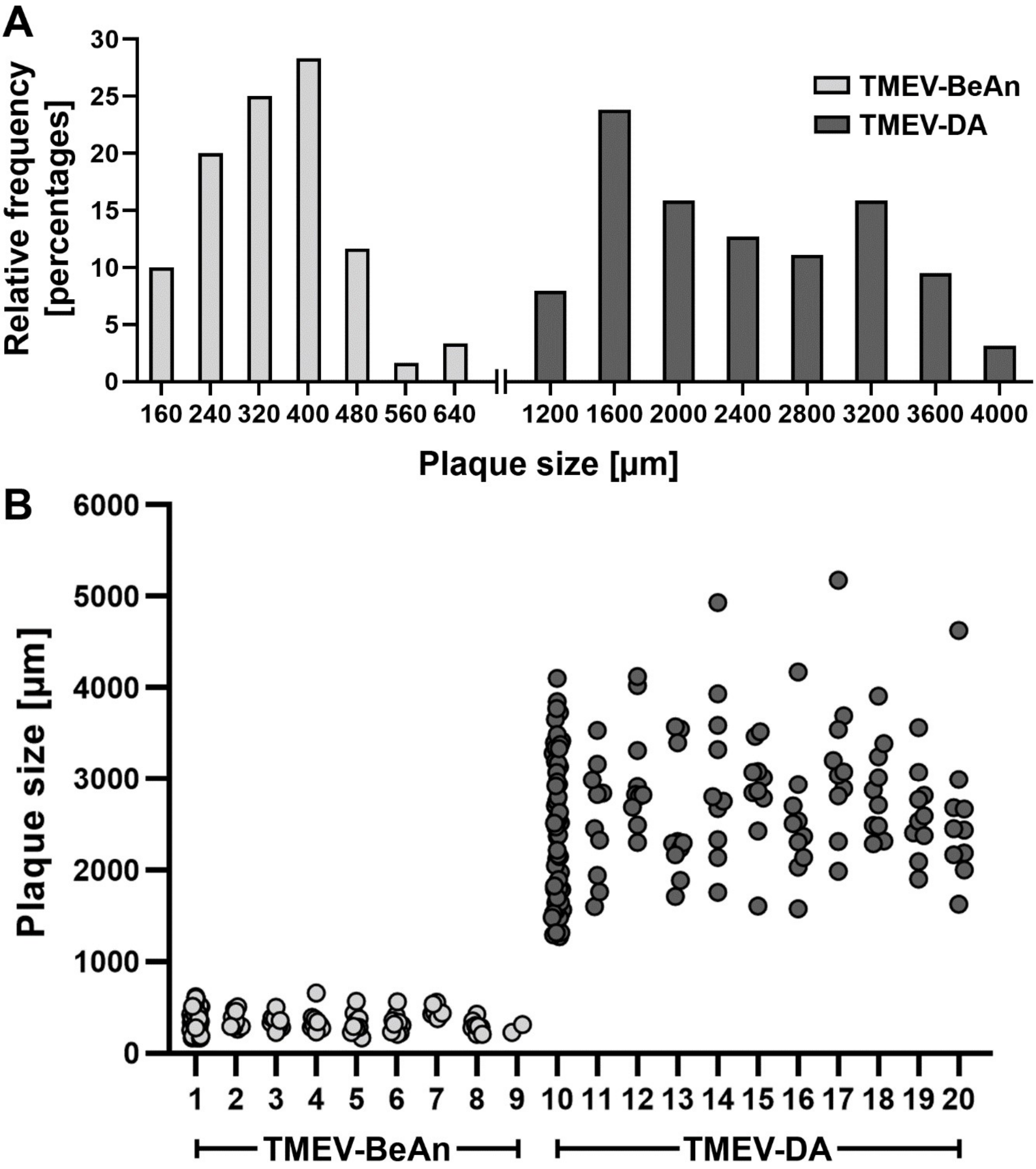

Supplemental Figure 3. Plaque size analysis of TMEV-BeAn and TMEV-DA strains used in the present study. (**A**) Frequency distribution analysis of plaques sizes caused by TMEV virus stocks used for infection. TMEV-BeAn induces relatively uniform small plaques, whereas TMEV-DA infection results in larger heterogeneous plaques. (**B**) Plaques sizes caused by TMEV-BeAn and TMEV-DA strains, which have been used for infection (#1 and #10, respectively) or re-isolated from infected mice (#2-9 and #11-20, respectively). Plaque sizes caused by original TMEV virus stocks and re-isolated viruses are similar.

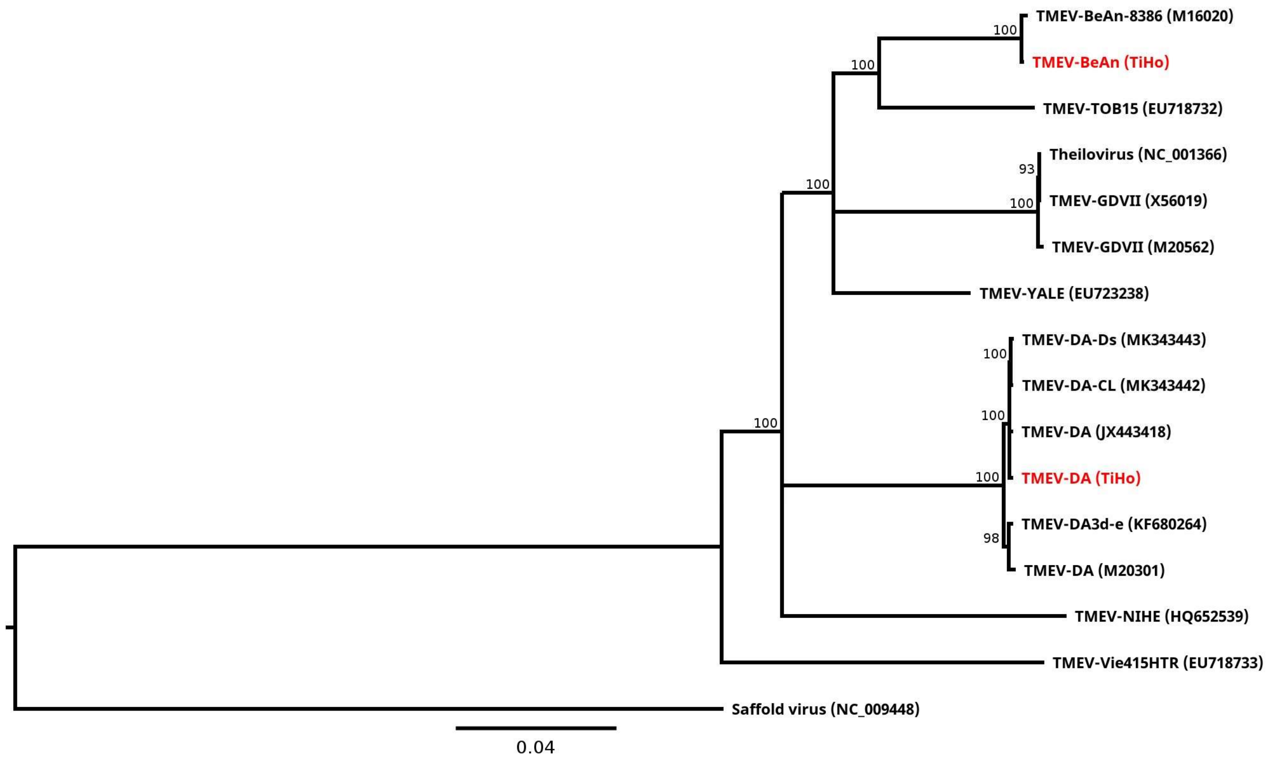

Supplemental Figure 4. Phylogenetic relationships between selected TMEV strains and other cardioviruses based on the nucleotide sequences of their complete genome. Sequences were aligned and phylogenetic analysis was performed as outlined in Material and Methods. Branch labels represent consensus support percentage of 1000 bootstrap replicates. The TMEV-BeAn and -DA strains used in the present study are marked in red. The scale bar indicates the horizontal distance of 0.04 substitutions per site.

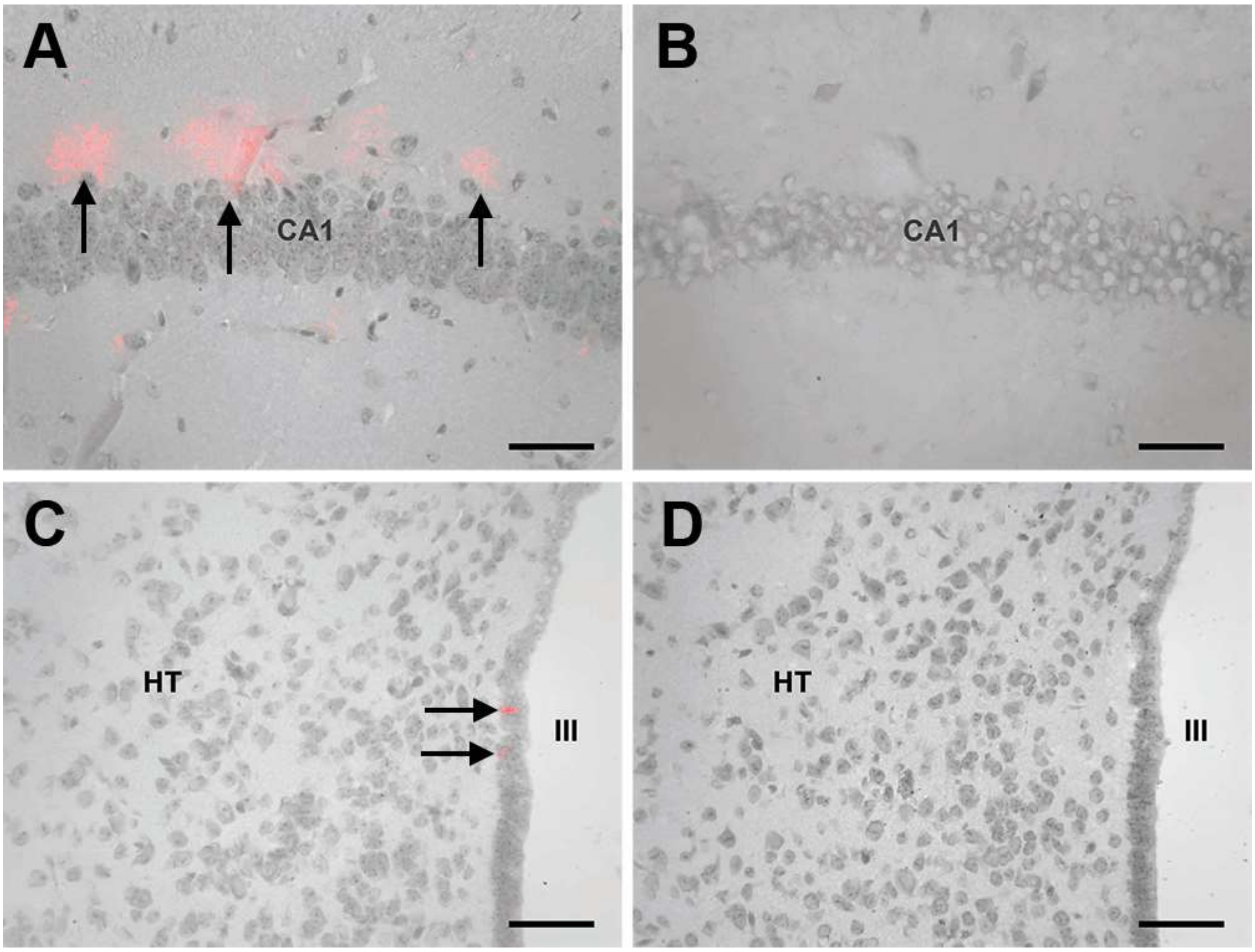

Supplemental Figure 5. Fluorescent in situ hybridization (FISH). Detection of IFN-β mRNA using „ViewRNA™ Probe Sets" (Affymetrix) in the brain of C57BL/6 wild type mice one day after infection with TMEV-BeAn. (**A**) Large amount of IFN-β (red) in the CA1 area of the hippocampus. (**B**) No signal in a serial section used as negative control. (**C**) Low amount of IFN-β (red) in ependymal cells of the third ventricle (III). HT = hypothalamus. (**D**) No signal in a serial section used as negative control. Bars = 50 µm.

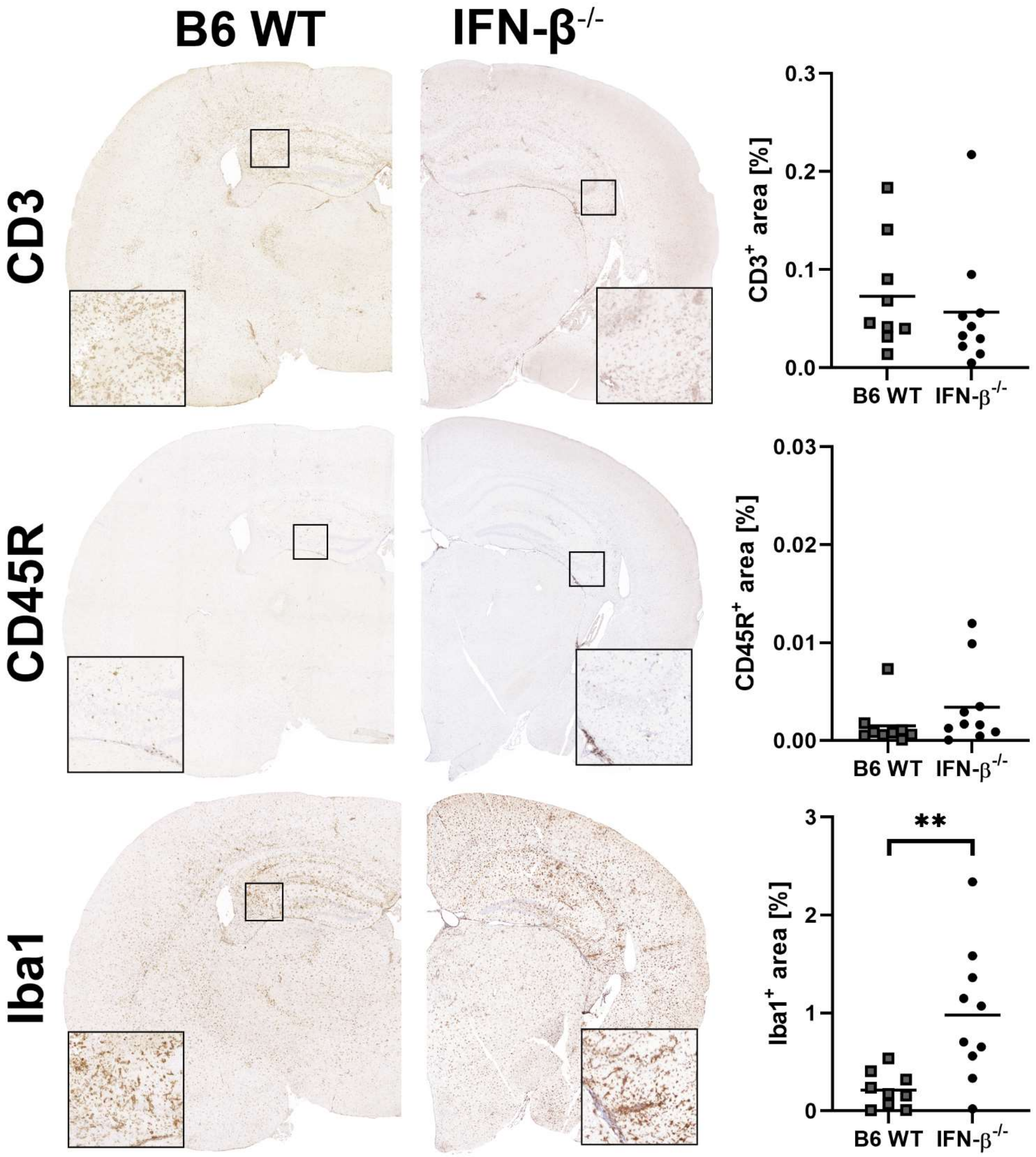

Supplemental Figure 6. IFN-β$^{-/-}$ mice show an increased number of Iba-1^{+} microglia/macrophages compared of *C57BL/6 wild type* (B6 WT) mice in the brain at 7/8 days after TMEV-DA infection. In contrast, IFN-β$^{-/-}$ deficiency did not change the infiltration of lymphocytes (CD3^{+} T cells and CD45R^{+} B cells). Immunohistochemistry (IHC) using the avidin-biotin complex (ABC) method. Percentages of immunopositive area were determined using morphometry of immunohistochemically stained brain sections (cerebrum with hippocampus, thalamus and hypothalamus). Mann-Whitney test: (** p=0.0021). Shown are all data points with means.

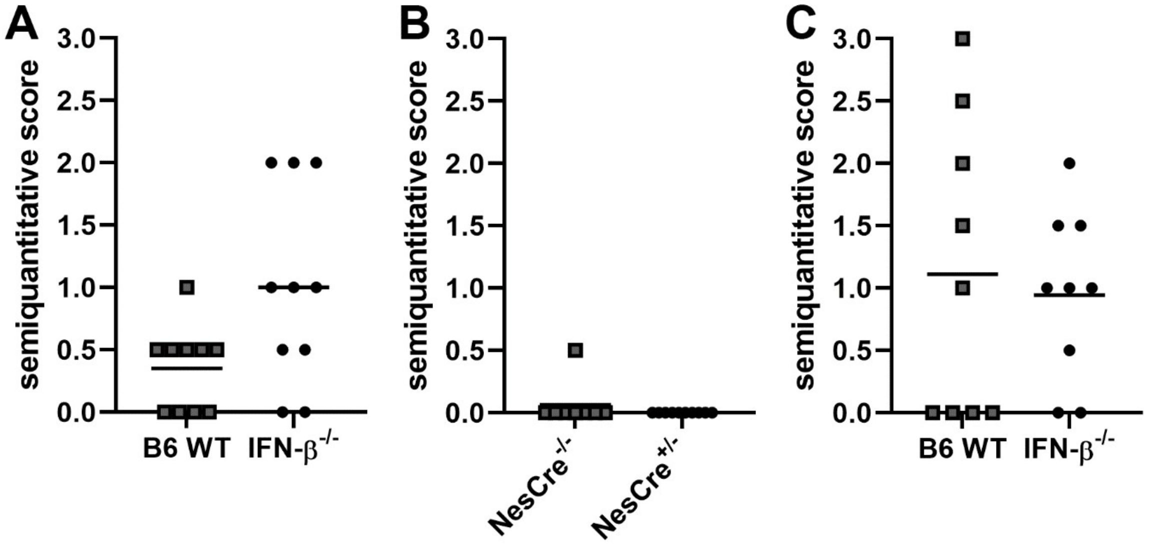

Supplemental Figure 7. Neuronal loss in the hippocampus of IFN-β$^{-/-}$ and *wild type* (B6 WT) mice (**A**) and NesCre$^{-/-}$ IFN-β$^{fl/fl}$ mice and NesCre$^{+/-}$ IFN-β$^{fl/fl}$ mice 14 days after infection with TMEV-BeAn (**B**) as well as IFN-β$^{-/-}$ and *wild type* (B6 WT) mice 7 days after infection with TMEV-DA (**C**). Semiquantitative analysis. Mann-Whitney tests did not show differences in neuronal loss between the respective knockout and control mice. Shown are all data points with means.

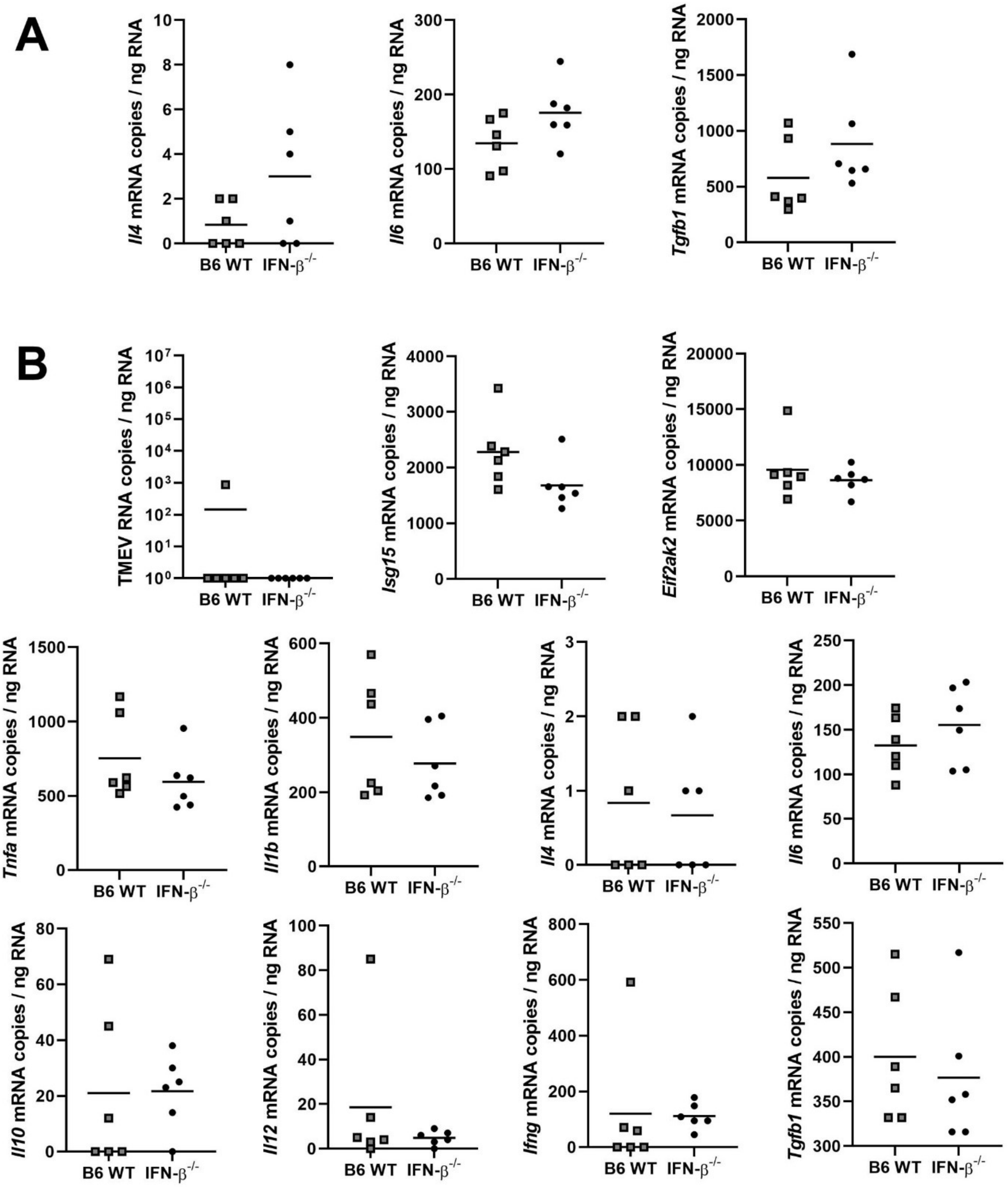

Supplemental Figure 8. RT-qPCR of the cerebrum at 14 and 98 *days post infection* (dpi). **(A)** Mann-Whitney tests did not detect differences in *Il4*, *Il6* and *Tgfb1* mRNA levels in the cerebrum of IFN-$\beta^{-/-}$ compared to *C57BL/6 wild type* (B6 WT) mice after TMEV-BeAn infection at 14 dpi. **(B)** Mann-Whitney tests did not show differences in the levels of TMEV RNA as well as *Isg15*, *Eif2ak2* (PKR), *Tnfa*, *Il1b*, *Il4*, *Il6*, *Il10*, *Il12*, *Ifng* and *Tgfb1* mRNA in the cerebrum of IFN-$\beta^{-/-}$ compared to B6 WT mice after TMEV-BeAn infection at 98 dpi. Shown are all data points with means.

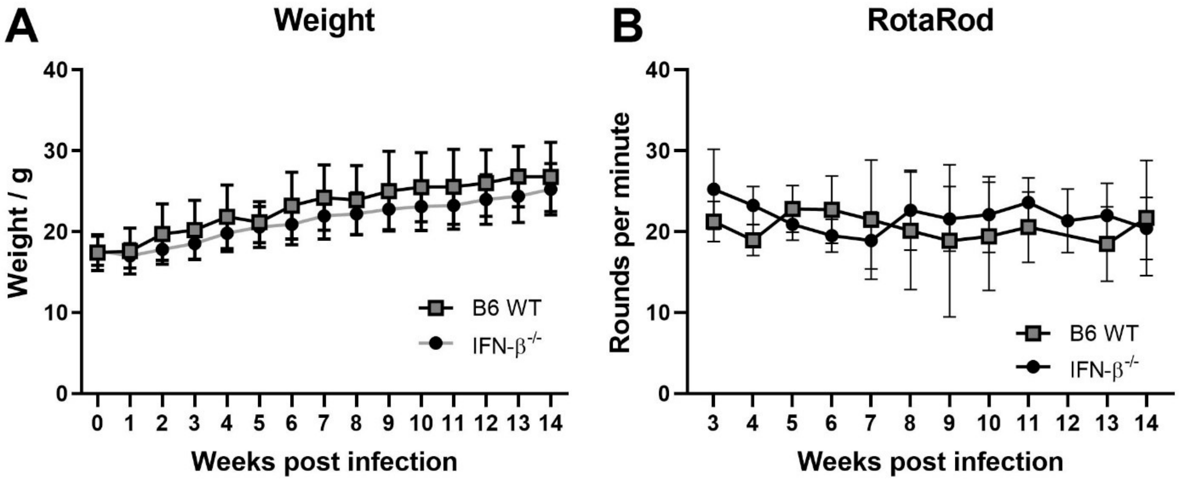

Supplemental Figure 9. Clinical data. TMEV-BeAn infected IFN-β$^{-/-}$ (n=10) and *C57BL/6 wild type* (B6 WT) mice (n=10) survive until the end of the investigation at 98 days post infection. Both strains continuously gain weight **(A)** and do not show impaired motor coordination in the RotaRod® performance test **(B)**.
